# Linkage Disequilibrium and Genome-Wide Association Mapping in Tetraploid Wheat (*Triticum turgidum* L.)

**DOI:** 10.1371/journal.pone.0095211

**Published:** 2014-04-23

**Authors:** Giovanni Laidò, Daniela Marone, Maria A. Russo, Salvatore A. Colecchia, Anna M. Mastrangelo, Pasquale De Vita, Roberto Papa

**Affiliations:** Consiglio per la Ricerca e la sperimentazione in Agricoltura, Cereal Research Centre, Foggia, Italy; Nanjing Forestry University, China

## Abstract

Association mapping is a powerful tool for the identification of quantitative trait loci through the exploitation of the differential decay of linkage disequilibrium (LD) between marker loci and genes of interest in natural and domesticated populations. Using a sample of 230 tetraploid wheat lines (*Triticum turgidum* ssp), which included naked and hulled accessions, we analysed the pattern of LD considering 26 simple sequence repeats and 970 mostly mapped diversity array technology loci. In addition, to validate the potential for association mapping in durum wheat, we evaluated the same genotypes for plant height, heading date, protein content, and thousand-kernel weight. Molecular and phenotypic data were used to: (i) investigate the genetic and phenotypic diversity; (ii) study the dynamics of LD across the durum wheat genome, by investigating the patterns of LD decay; and (iii) test the potential of our panel to identify marker–trait associations through the analysis of four quantitative traits of major agronomic importance. Moreover, we compared and validated the association mapping results with outlier detection analysis based on population divergence. Overall, in tetraploid wheat, the pattern of LD is extremely population dependent and is related to the domestication and breeding history of durum wheat. Comparing our data with several other studies in wheat, we confirm the position of many major genes and quantitative trait loci for the traits considered. Finally, the analysis of the selection signature represents a very useful complement to validate marker–trait associations.

## Introduction

Determination of the genetic basis of agronomic traits has been one of the major scientific challenges in the process of crop improvement. In plants, linkage mapping is currently the most common approach for detection of quantitative trait loci (QTLs) that correspond to agronomic traits. In linkage mapping, linkage disequilibrium (LD) is generated by establishing populations that are derived from bi-parental crosses, and co-segregation of alleles of mapped marker loci and phenotypic traits allows the identification of linked markers. In durum and bread wheat, QTL mapping has been used frequently to dissect out the genetic architecture of agronomic and quality traits using doubled haploids, recombinant inbred lines, and backcross populations. Several main-effect QTLs have been identified for yield [1]–[4], grain protein content [5]–[10], thousand-kernel weight [6]–[8], disease resistance [11]–[14], and flowering time [15]–[18]. Due to the restricted number of meiotic events that are captured in a biparental mapping population, the genetic resolution of QTL mapping often remains confined to a range of 10 cM to 30 cM [19]–[20]. Moreover, linkage analysis can only sample a small fraction of all of the possible alleles in a population from which the parents originated.

To overcome the constraints inherent to linkage mapping, a complementary strategy is based on the correlation of genotype with phenotype in domesticated and natural populations, and this is commonly referred to as association mapping (or LD mapping), where the focus turns from families to populations. The principle that underlies this approach is that LD tends to be maintained between linked loci over many generations. High LD is expected between loci in tight linkage, while recombination should have eliminated LD between unlinked loci [21]. Linkage disequilibrium mapping exploits all of the historical recombination events that have occurred in the population from the origin of the marker–trait association [22]–[25]. Linkage disequilibrium mapping was first introduced in genetic mapping studies in human [26]–[27], and was then later considered for plant studies [19], [28]–[33].

A major problem of association mapping is the high probability of false-positive associations between a marker and a trait (type I error) [34]. However, for LD mapping to reduce the risk of false positives, more complex statistical tools are required than those used for linkage mapping. The Bayesian model-based cluster method was developed to infer population structures in complex pedigree populations [35], whereby a set of random markers is used to estimate the population structure (*Q*), which is incorporated into a general linear model (GLM) for testing associations. This has been widened to a mixed linear model (MLM), which also includes the kinship relationships of the samples, and offers improved control of type I error rates, which accounts for the population structure and relatedness in association mapping [36]. In addition, a useful strategy to reduce the risk of false positives is the use, when appropriate, of independent validation of the detected associations (e.g., by linkage mapping). In some cases, to validate the results of mapping studies and to identify loci/genes or genomic regions that are involved in the genetic control of important adaptive traits, a promising approach is to combine the association mapping with an analysis of the signature of selection based on the structure of the molecular diversity, without the use of phenotypic data [37]–[38]. The success of LD mapping depends on the quality of the phenotypic data, the population size, and the degree of LD in a population [28]–[29]. The level of LD that characterises the species and the population used [22], [25], [30], [39] is a critical aspect for the planning of association and/or population genomics studies, because this determines the resolution of the association mapping. When LD is low, a candidate gene approach is preferred, because in this case, too many markers will be needed to perform a whole genome scan to cover the variation in the entire genome. On the other hand, when LD is moderate/high, a whole genome scan can be more appropriate. An ideal situation would be to use different populations distinguished by variable LD levels.

Durum wheat (*Triticum turgidum* ssp. *turgidum* var. *durum*) is of significant commercial importance, because of its end-use products: pasta, cous-cous, bread and bulgur. For this species, the combined effects of domestication and modern plant breeding have contributed to its greatly reduced genetic diversity in comparison to the wild forms [40]–[43]. In this context, landraces, wild forms (*Triticum turgidum* ssp.), and other related wild species might have crucial roles in breeding programmes, given their wide variability in terms of their many phenological, morphological, abiotic, biotic and quality traits. Here, the data used are from an association-mapping panel that is composed of 230 tetraploid wheat accessions (elite varieties, wild and domesticated accessions) that have been fingerprinted according to 26 simple sequence repeats (SSRs) and 970 diversity array technology (DArT) markers, and evaluated for plant height (PH), heading date (HD), protein content (PC), and thousand kernel weight (TKW). These data are used to: (i) investigate the genetic and phenotypic diversity; (ii) study the dynamics of LD across the durum wheat genome, by investigating the patterns of LD decay; and (iii) test the potential of our panel through the analysis of four quantitative traits of major agronomic importance, to identify marker–trait associations (MTAs) using both GLMs and MLMs.

## Materials and Methods

### Plant material and genotyping

This study evaluated a tetraploid wheat (*T*. *turgidum* L., 2n = 4x = 28; AABB genome) collection of 230 inbred lines (Table 1), as reported by Laidò et al. [43]. The whole collection consists of 128 durum wheat varieties plus 102 wild and domesticated accessions. These durum wheat varieties are indigenous and exotic landraces, and modern cultivars, most of which are materials that are representative of the Italian durum wheat breeding programmes over the last 100 years. Table 1 gives the number of accessions for each sub-species, and Laidò et al. [43] provided a detailed list of the genotypes (number/name, year of release, country, pedigree). The whole tetraploid wheat collection, which also includes wild and domesticated accessions, was assembled to cover the phenotypic variability for the breeding traits that were evaluated in the present study. The LD analysis was conducted on the whole collection, on the 128 durum wheat varieties (henceforth referred to as the *durum* sub-sample), and on two subgroups identified through the population structure analysis (Q1, Q2) as reported by Laidò et al. [43].

**Table 1:** Number of accessions for each subspecies included in the whole wheat collection and in the Q1 and Q2 subgroups.

Tetraploid wheat classification (AABB)		Naked/hulled	N° of	Subgroups (Structure K 2 - 26 SSR)	
van Slageren (1994)	MacKey (1988)	kernel	accessions	Q1	Q2
*T. turgidum* ssp. *turgidum* convar. *durum*	*T. turgidum* ssp. *durum*	Naked	128	122	6
*T. turgidum* ssp. *turgidum* convar. *turanicum*	*T. turgidum* ssp. *turanicum*	Naked	20	3	17
*T. turgidum* ssp. *turgidum* convar. *polonicum*	*T. turgidum* ssp. *polonicum*	Naked	20	4	16
*T. turgidum* ssp. *turgidum* convar. *turgidum*	*T. turgidum* ssp. *turgidum*	Naked	19	-	19
*T. turgidum* ssp. *carthlicum*	*T. turgidum* ssp. *carthlicum*	Naked	12	-	12
*T. turgidum* ssp. *dicoccum*	*T. turgidum* ssp. *dicoccum*	Hulled	19	-	19
*T. turgidum* ssp. *dicoccoides*	*T. turgidum* ssp. *dicoccoides*	Hulled	12	-	12
**Total**			**230**	**129**	**101**

As described in Laidò et al. [43], for each accession, the leaf tissue of the plants that represented the prevalent biotype of each accession was used for DNA extraction, and the whole collection was genotyped according to the 26 SSR and 970 DArT markers used. Thus, in the present study, we used the dataset developed by Laidò et al. [43], and we exploited the mapping information obtained by Marone et al. [44], which was used to determine the map positions of the SSR and DArT markers.

Alleles that occurred at low frequency (*f*<0.05) were excluded from all of the present analysis. The genetic diversity, or heterozygosity (*H*), was investigated within the whole collection and within the *durum* sub-sample for each chromosome, by calculation of the unbiased estimator of genetic diversity (H_E_ [45]). To measure the relative loss of genetic diversity in the whole collection *versus* the *durum* sub-sample, according to Vigoroux et al. [34], we used the parameter ΔGD = 1-(H_E(wc)_/H_E(durum)_), where H_E(wc)_ and H_E(durum)_ are the genetic diversity for the whole collection and for the *durum* sub-sample, respectively.

### Linkage disequilibrium

The LD between the mapped DArT markers on the durum wheat consensus linkage map constructed by Marone et al. [44] was assessed for each sample only on markers that showed frequencies ranging from 0.05 to 0.95. The LD (allele frequency correlation, *r^2^*) estimates [46] between marker pairs were obtained using the TASSEL 3.0.115 software. The significance of the pair-wise LD (*P* values) was computed using 1,000 permutations. The LD statistics were calculated per chromosome, and subsequently aggregated over all of the chromosomes of the A and B genomes. The LD was calculated separately for loci on the same chromosome (intrachromosomal pairs) and for unlinked loci (interchromosomal pairs). From the unlinked loci, a critical value of *r^2^* was estimated following Breseghello and Sorrells [47], by root transforming the *r^2^* values and taking the 95% percentile of this distribution as the threshold beyond which the LD is probably caused by a real physical linkage. The intrachromosomal *r^2^* values were plotted against the genetic distance, and a smooth line was drawn using second-degree locally weighted polynomial regression (LOESS) [48], obtained with the statistical programme *R* (http://www.r-project.org), to determine at which distance the LOESS curve intercepts the critical *r^2^*, to see how rapidly the LD decay occurs. The LD analysis for the mapped markers on the durum wheat consensus linkage map was performed separately for the whole collection, the *durum* sub-sample, and the two subgroups identified with the genetic structure analysis (Q1, Q2). The LD was also estimated among the unmapped MTAs and mapped markers, although only for the whole collection.

### Phenotypic evaluation and association analysis

To test the potential of our panel to perform association studies, we investigated four quantitative traits of major agronomic importance. In particular, the tetraploid wheat collection of 230 inbred lines was evaluated for PH, HD, PC and TKW in a field trial conducted in Foggia (41°28 N, 15°32 E; altitude, 75 m a.s.l.) over one season (2007/2008). The field trial was sown on 20 December, 2007, in clay-loam soil (typic Chromoxerert), in a randomised block design with four replicates. The plots were 2.0 m×1.5 m, with a seed density of 350 germinated seeds/m^2^. According to the standard agronomic practices in the study area, fertiliser applications were made at pre-sowing (36 kg/ha N, 90 kg/ha P_2_O_5_, as ammonium biphosphate) and top dressing (52 kg/ha N, as urea) at Zadoks growth stage 2.2 and 3.1 [49], respectively. The plants were harvested after physiological maturity, on 10 June, 2008, and the grain was ground into whole meal in an experimental mill (Tecator Cyclotec1093), and used for the grain quality analysis.

The HD is reported as the number of days from 1 April, 2008, until the ears of *ca*. 50% of the tillers had emerged from the flag-leaf sheaths by approximately half of their length (growth stage 55 of the Zadoks scale; [49]). The PH (cm) was measured during the milk waxy maturation stage, when the maximum height was achieved, from the ground to the ear tip (excluding awns), on five main culms per plot. Following determination of the grain nitrogen content according to the standard Kjeldhal method, the PC was then calculated by multiplying the Kjeldhal N value by 5.7, which was then expressed as a percentage on a dry-weight basis. The TKW (g) was calculated from the mean weight of three independent samples of 1000 grains for each plot.

The TASSEL software, version 3.0.115, was used to calculate the associations between the markers and each trait in turn. Both GLMs and MLMs were used in the association analysis for the whole collection and for the *durum* sub-sample. For the GLMs, genotypic data, phenotypic data and the *Q* matrix were integrated as covariates to correct for the effects of population substructure, which were determined for the whole collection (K = 2 with SSR markers) and for the *durum* sub-sample (K = 3 with SSR markers). In the MLMs, the Kinship matrix (*K* matrix) was used in addition to the genotypic data, phenotypic data and *Q* matrix, to correct for both population and family structure. In total, the genotypic score was available for 984 loci in the whole collection (958 DArT markers and 26 SSR loci), and for 871 markers in the *durum* sub-sample (845 DArT markers and 26 SSR loci). To generate a marker similarity matrix containing all of the lines (*K* matrix) using the TASSEL software, the trimmed marker datasets were used. TASSEL calculates the kinship as the proportion of alleles shared between each pair of lines. Once this matrix was calculated, the numbers were rescaled, so that they were between 0 and 2 [38]. The critical *P* values for the assessment of the significance of the MTAs were calculated separately for each trait, based on a false discovery rate (FDR) of 0.05, which is defined as the expected proportions of the true null hypotheses that are rejected [50].

To compare our data with data obtained in other studies, we considered the most recently published information on genes and QTL mapping for the traits (PH, HD, PC, TKW). All of the significant MTAs, identified using the MLM model, located on the durum wheat consensus map within short map intervals (5–10 cM) were grouped into a single putative QTL.

As reported in Laidò et al. [43], the population differentiation was also assessed by analysis of molecular variance (AMOVA) using the ARLEQUIN software, version 3.5 [51]. Through the *Fst*-outlier detection method, and using 100,000 simulations, 211 loci under selection were identified, of which 109 markers were mapped on the durum wheat consensus map and used to validate the MTAs, or the region containing the MTAs, that were identified for each trait by association mapping.

## Results

### Diversity and linkage disequilibrium

From the 970 polymorphic DArT markers, 592 were genetically mapped on the durum wheat consensus linkage map constructed by Marone et al. [44]. Of these, none were excluded, because the minor allele frequencies in the whole collection and in the *durum* sub-sample were >0.05. Although these only represented half of the markers used in the present study, they covered an estimated 2,247 cM, which represents approximately 73.5% of the durum wheat consensus map [44]. These DArTs were distributed over all of the chromosomes, with an average spacing of 4.0 cM (Table 2). The distribution of DArTs varied within and among the chromosomes, with a maximum of 75 markers on chromosome 3B, and a minimum of 12 markers on chromosome 5A (Table 2).

**Table 2:** DArT markers coverage, distribution, index of diversity and loss of diversity among the whole collection and *durum* sub-sample, with the distribution and index of diversity in the two Q groups identified in the whole collection.

Chromosome	cM	N° of markers	Marker coverage	H_E_ Index of whole collection	H_E_ Index of *durum* sub-sample	Loss of diversity	Subgroups (Structure K 2 - 26 SSR)			
							Q1		Q2	
							N° of markers	H_E_ Index	N° of markers	H_E_ Index
1A	37.1	25	1.9	0.40	0.31	0.23	24	0.31	25	0.42
2A	213.4	33	5.5	0.44	0.40	0.09	33	0.40	32	0.38
3A	83.6	20	3.8	0.43	0.38	0.12	20	0.38	20	0.43
4A	96.1	57	1.7	0.40	0.39	0.03	55	0.39	55	0.34
5A	67.2	12	5.2	0.44	0.43	0.02	12	0.44	11	0.38
6A	160.7	55	2.9	0.45	0.42	0.07	55	0.42	55	0.45
7A	219.4	35	6.3	0.41	0.40	0.02	35	0.40	32	0.35
1B	216.5	53	4.1	0.41	0.40	0.02	51	0.40	49	0.38
2B	225.7	53	4.3	0.45	0.44	0.02	52	0.44	51	0.37
3B	240.7	75	2.8	0.44	0.40	0.09	70	0.40	75	0.37
4B	107.8	16	6.7	0.40	0.37	0.08	16	0.37	16	0.41
5B	218.0	35	5.4	0.38	0.33	0.13	32	0.33	35	0.36
6B	140.1	60	2.3	0.40	0.40	0.00	59	0.41	55	0.35
7B	220.8	63	3.5	0.41	0.42	0.00	63	0.42	54	0.34
**Genome A**	877.5	237	3.9	0.42	0.39	0.08	234	0.39	230	0.39
**Genome B**	1,369.6	355	4.1	0.41	0.39	0.05	343	0.40	335	0.37
**Total**	2,247.1	592	4.0	0.42	0.39	0.06	577	0.39	565	0.38

To understand the overall genetic diversity among the whole collection and the *durum* sub-sample, we evaluated the genetic diversity (H_E_) and the loss of diversity for each chromosome (Table 2). The results obtained with 592 DArT markers indicate that the diversity of the *durum* sub-sample represents 94% of the whole genome, thus with a loss of diversity of 6%, with H_E_ of 0.42 and 0.39 for the whole collection and for the *durum* sub-sample, respectively. Chromosome 1A shows the highest values of loss of diversity (ΔH_E_ = 0.23), with H_E_ values for the whole collection and the *durum* sub-sample, of 0.40 and 0.31, respectively (Table 2).

Linkage disequilibrium analysis was performed for the whole collection, the *durum* sub-sample, and each of the two subgroups identified with the genetic structure analysis using the SSR markers (Q1, Q2). Pairwise LD was estimated using the squared-allele frequency correlations (*r* ^2^). As reported in Figure S1, we observed that the LD varies along the chromosomes, with regions of high LD interspersed with regions of low LD. The LD pattern in the whole collection was assessed based on the 174,936 pairwise combinations of 592 DArTs. Interchromosomal LD was investigated for the set of 162,635 pairs of non-syntenic loci (i.e., loci located on different chromosomes), with an average *r* ^2^ of 0.02 (Table 3). Based on permutation analysis, the portion of DArT pairs that showed significant LD was 22.7%, at *P*<0.01.

**Table 3:** Interchromosomal LD for the whole collection, the *durum* sub-sample, and the Q1 and Q2 groups, and for genomes A and B.

Dataset	N° of markers	Total N° pairs	Mean *r^2^* for all pairs	N° significant pairs	Significant pairs (%)	N° pairs in complete LD
**Whole collection**	592	162,635	0.02	36,966	22.7	0
***Durum*** ** sub-sample**	592	162,635	0.03	23,417	14.4	0
**Q1**	577	154,455	0.03	24,771	16.0	0
**Q2**	565	148,293	0.04	20,489	13.8	0
**Genome A**	237	24,067	0.02	5,469	22.7	0
**Genome B**	355	54,433	0.02	12,324	22.6	0

Mean allele frequency correlations (*r^2^*) for all pairs, number (N°) of pairs and percentage (%) significant in LD (*P*<0.01), N° of pairs in complete LD (*r^2^* = 1).

**Figure 1:**
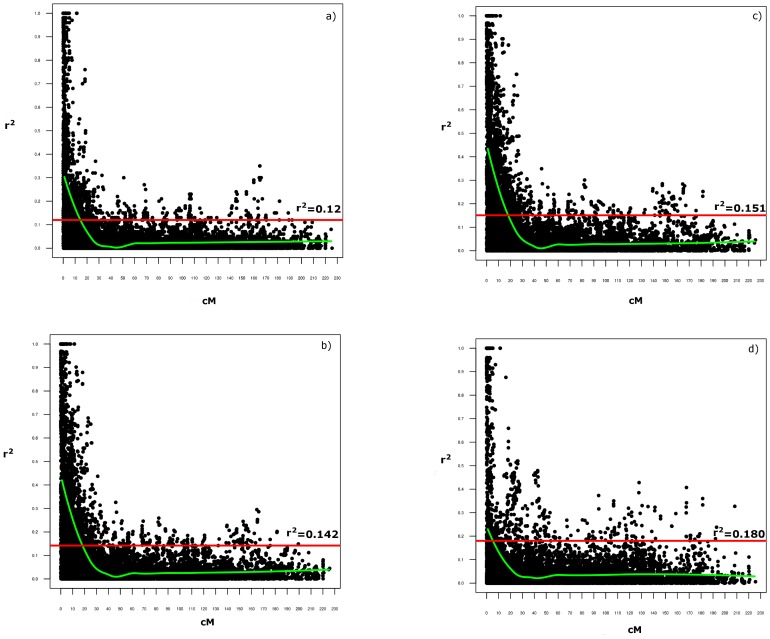
Overview of the LD parameter *r^2^*of the intrachromosomal pairs in the whole collection (Pop) (a), in the *durum* sub-sample (b), and in the two Q groups, Q1 (c) and Q2 (d). The scatterplots show the distributions of the LD parameter *r^2^* according to the genetic distance. The horizontal line indicates the 95% percentile of the distribution of the unlinked *r^2^*, which gives the critical value of *r^2^*.

The plots of the LD estimates (*r^2^*) as a function of genetic distance in centiMorgans (cM) indicated a clear decay of the LD with genetic distance, and also suggested that the LD is dependent on the population structure (Figure 1). As expected, pairs of physically linked (syntenic) loci (12,301 pairs in total, with an average *r* ^2^ of 0.08) showed LD estimates that were largely influenced by their physical distances (Table 4; Table S1). Due to the presence of significant LD effects that would be determined by population structure, assuming an absence of epistatic selection between the loci on different chromosomes, the *ad-hoc* critical thresholds for the LD estimates between syntenic loci were obtained from the distribution of the non-syntenic loci. According to Breseghello and Sorrells [47], the 95^th^ percentile of the LD estimate distribution was used as the critical threshold to better discriminate the LD that was most likely due to physical linkage. In the whole collection, a critical value of *r* ^2^ (basal LD) was calculated from the interchromosomal LD analysis (*r* ^2^ = 0.120), beyond which the LD is assumed to be caused by physical linkage (Figure 1a). Four classes of marker pairs were defined on the basis of the map positions determined by Marone et al. [44]: class 1 (tight linkage; distance, <10 cM); class 2 (moderate linkage; distance, 10–20 cM); class 3 (loosely linked; distance, 20–50 cM); and class 4 (independent pairs; distance, >50 cM). The percentages of significant loci pairs decreased with the distance between the loci; 77.7% of the 2,717 pairs of the tightly linked markers (within 10 cM) showed significant LD at *P*<0.01, with an average *r* ^2^ of 0.26 (Table 4; Table S1). For the remaining classes, the LD varied from 52.8% (class 2) to 25.8% (class 4), decreasing at increasing distances. Figure 1a shows the scatterplot of the distributions of the *r* ^2^ values as a function of the genetic distances between the syntenic loci pairs for the whole collection. The point at which the LOESS curve intercepts the critical *r* ^2^ was determined as the average LD decay of the population. Based on these criteria, the intrachromosomal LD decayed between 5 cM and 20 cM for the individual chromosomes (considering only chromosomes with >50 markers) (Figure S1) and the average LD decay of the whole genome was 14 cM (Figure 1a). Also, for the portion of *r* ^2^ values that exceeded the basal LD level, the LD decreased from 60.7% of significant pairwise comparisons (class 1; mean *r* ^2^ = 0.41) to less than 6.0% (class 4; mean *r* ^2^ = 0.13). All of the loci pairs that are in complete LD are spaced at a genetic distance <10 cM, except for two loci pairs at genetic distances from 10 cM to 20 cM.

**Table 4:** Overview of LD in the intrachromosomal pairs for the whole collection, the *durum* sub-sample, and the Q1 and Q2 groups, and for genomes A and B.

Dataset	N° total pairs	Mean *r^2^* of all pairs	N° significant pairs	Significant pairs (%)	N° physically linked pairs	Physically linked pairs (%)	Mean r^2^ for physically linked pairs	N° pairs in complete LD
**Whole collection**	12,301	0.08	5,066	41.2	2,609	21.2	0.31	192
***Durum*** ** sub-sample**	12,301	0.12	4,570	37.2	3,643	29.6	0.36	279
**Q1**	11,721	0.13	4,618	39.4	3,051	26.0	0.42	280
**Q2**	11,037	0.08	2,246	20.3	1,373	12.4	0.42	201
**Genome A**	3,899	0.12	1,773	45.5	1,050	26.9	0.34	99
**Genome B**	8,402	0.07	3,293	39.2	1,559	18.6	0.30	93

Mean allele frequency correlations (*r^2^*) for all pairs, number (N°) of pairs and percentage (%) significant in LD (*P*<0.01), N° and % of physically linked pairs (*r^2^*>critical *r^2^*), N° of pairs in complete LD (*r^2^* = 1).

### Patterns of linkage disequilibrium within subgroups and the durum sub-sample

An overview of the interchromosomal LD in the two subgroups and in the *durum* sub-sample is given in Table 3. The mean of the interchromosomal LD is much lower than that observed for the intrachromosomal LD. The Q1 and Q2 groups that were obtained from the analysis of the population structure show higher *r* ^2^ and lower numbers of significant pairs, compared to the whole collection. The Q1 group shows a mean *r* ^2^ and number of significant pairs similar to the *durum* sub-sample (Table 3). At the intrachromosomal level, the LD pattern for the *durum* sub-sample was assessed based on 12,301 pairwise combinations, with an average *r* ^2^ of 0.12 (Table 4; Table S1). Based on permutation analysis, the portion of the DArT pairs that show significant LD is 37.2%. The amount of significant LD and the *r* ^2^ in the *durum* sub-sample and in these two subgroups are listed in Table 4 and Table S1. In all of the classes, the values for the *durum* sub-sample and for the Q1 group are very close to the values for the whole collection, in terms of the number of pairs that show significant LD, whereas the mean *r* ^2^ are much higher in all of the classes based on the genetic distances. Furthermore the number of pairs in the total LD is much higher in the *durum* sub-sample and the Q1 group (279 and 280 loci pairs, respectively), compared to 192 in the whole collection and 201 in the Q2 group (Table 4; Table S1). The pairs in total LD have mean distances of 0.80 cM and 0.89 cM in the *durum* sub-sample and the Q1 group, respectively, whereas in the Q2 group and in the whole collection, this spans over mean distances of 0.61 cM and 0.53 cM, respectively. At 0.151 and 0.180, the critical *r* ^2^ for the Q1 (Figure 1c) and Q2 (Figure 1d) groups, respectively, are higher compared to the whole collection (critical *r* ^2^ = 0.120) (Figure 1a) and the *durum* sub-sample (critical *r* ^2^ = 0.142) (Figure 1b). The *durum* sub-sample and the Q1 group follow the same pattern in the decay of the LD, and the mean LD decays at ∼18 cM (Figure 1b, c); the Q2 group falls below the 0.180 threshold from a distance of ∼5 cM (Figure 1d).

### Phenotypic data

The accessions at the experimental farm of CRA-CER Foggia, Italy, were evaluated for the key traits of PH, HD, PC and TKW. For all of these traits investigated, a clear quantitative genetic basis was evident, both for the whole collection and for the *durum* sub-sample (see phenotypic distributions in Figures S2, S3). The ANOVA analysis shows that the genotypic variance is significantly >0 for all of the traits (P<0.001). The hereditabilities ranged from 0.67 to 0.91 in the whole collection, and 0.29 to 0.84 in the *durum* sub-sample, which indicates the robustness of the data and the low error rate. All of the statistic parameters for the traits are given in Table 5, for PH, HD, PC and TKW.

**Table 5:** Statistical estimations of all of the traits for the whole collection and the *durum* sub-sample, including the heritability (H^2^) and least significant different (LSD).

Trait	Dataset	Mean	Minimum	Maximum	H^2^	LSD (0.05)
Plant height (cm)	Whole collection	97.6	62.5	151.3	0.91	11.0
	*Durum* sub-sample	81.2	62.0	133.8	0.84	8.4
Heading date (days)	Whole collection	36.2	22.0	50.3	0.89	3.1
	*Durum* sub-sample	32.1	22.0	46.7	0.81	3.1
Protein content (%)	Whole collection	18.7	13.8	25.1	0.67	1.6
	*Durum* sub-sample	18.1	15.7	20.4	0.29	1.7
Thousand kernel weight (g)	Whole collection	45.3	21.8	66.8	0.85	4.8
	*Durum* sub-sample	46.2	31.5	59.6	0.64	5.2

### Association mapping

The marker–phenotype association analysis was based on the polymorphisms present in 26 SSRs, and for DArTs, at 958 and 845 loci in the whole collection and in the *durum* sub-sample, respectively. Each locus was characterised by the presence of at least one non-rare allele, with a frequency between 0.95 to 0.05.

Both GLM and MLM analysis were compared for all of the traits. A cut-off of 0.05 for the false discovery rate [50] was used to identify the MTAs. Each MTA represents a significant marker–trait association, and for each marker, a maximum of “n” MTAs can be identified, where “n” is the number of traits under study. The number of significant MTAs with the GLM is much greater than with the MLM (Table 6). Indeed, for the whole collection, there were 1,296 and 221 MTAs for the GLM and MLM, respectively, while in the *durum* sub-sample, there were 464 and 191 MTAs identified with the GLM and MLM, respectively. Thus, with the MLM, the MTAs were reduced by 82.9% in the whole collection, and by 58.8% in the *durum* sub-sample. There are 161 MTAs in the whole collection and 160 in the *durum* sub-sample that are shared by the two models (Table 6). The number of unique (significant only for one model) MTAs for the GLM was 87.6% (whole collection) and 65.5% (*durum* sub-sample), compared to only 27.1% and 16.2%, respectively, for the MLM (Table 6).

**Table 6:** Comparison of the two models for the calculation of the associations between the mapped and unmapped markers, and the traits, for the whole collection and the *durum* sub-sample.

Trait	Dataset	N° Total sign.	N° GLM sign.	N° MLM sign.	Sign. in GLM and MLM n (%)	N° GLM unique	N° MLM unique	Unique GLM (%)	Unique MLM (%)
Plant height	Whole collection	407	392	45	30 (7.4)	362	15	88.9	3.7
	*Durum* sub-sample	146	135	41	30 (20.5)	105	11	71.9	7.5
Heading date	Whole collection	256	250	61	55 (21.5)	195	6	76.2	2.3
	*Durum* sub-sample	120	117	42	39 (32.5)	78	3	65.0	2.5
Protein content	Whole collection	222	197	60	35 (15.8)	162	25	73.0	11.3
	*Durum* sub-sample	119	114	50	45 (37.8)	69	5	58.0	4.2
Thousand kernel weight	Whole collection	471	457	55	41 (8.7)	416	14	88.3	3.0
	*Durum* sub-sample	110	98	58	46 (41.8)	52	12	47.3	10.9

GLM  =  general linear model (Q matrix), MLM  =  mixed linear model (Q matrix + kinship matrix), sign.  =  significant with *P*<0.05.

### Trait mapping

**Figure 2:**
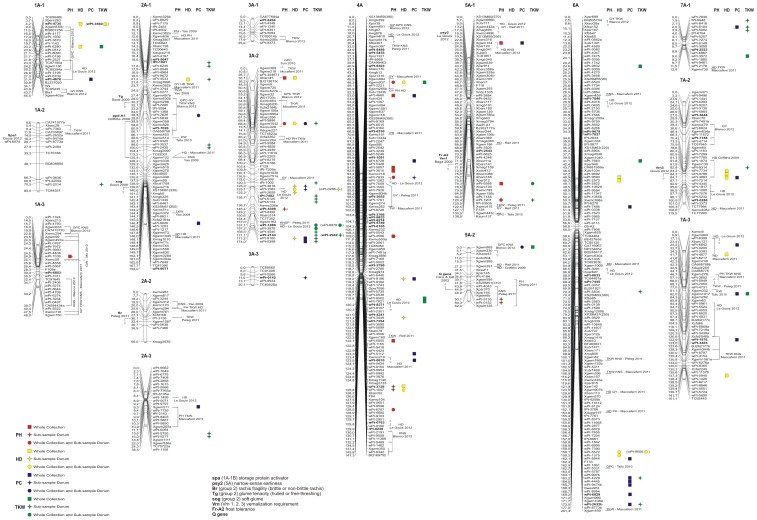
Genome A: Durum wheat consensus linkage map (reported in Marone et al. [44]) of the chromosome positions of significant markers associated with plant height (red), heading date (yellow), protein content (blue) and thousand kernel weight (green). Right side: squares, MTAs in the whole collection; stars, MTAs in the *durum* sub-sample; circles, MTAs in both. DArT markers putatively under selection are indicate in bold, while the unmapped MTA are given in brackets.

**Figure 3:**
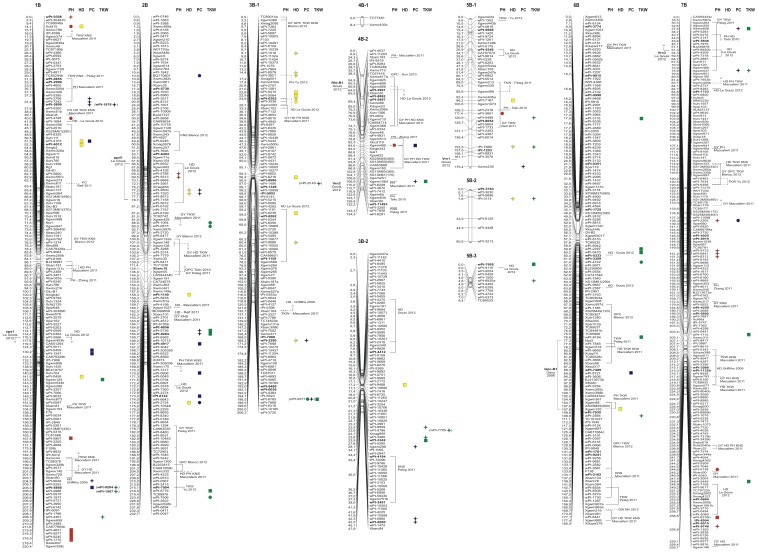
Genome B: Durum wheat consensus linkage map (reported in Marone et al. [44]) of the chromosome positions of significant markers associated with plant height (red), heading date (yellow), protein content (blue) and thousand kernel weight (green). Right side: squares, MTAs in the whole collection; stars, MTAs in the *durum* sub-sample; circles, MTAs in both. DArT markers putatively under selection are indicate in bold, while the unmapped MTA are given in brackets.

Regarding the significant associations, all of the data for each MTA are shown in Table S2. For all of the traits, only the significant MTAs mapped on the durum wheat consensus map that were derived from the MLM are shown in Figures 2 and 3, and reported in Tables 7, 8, 9, 10, for PH, HD, PC and TKW, respectively.

**Table 7:** Representative markers significantly associated with the plant height trait, for the whole collection (normal), the *durum* sub-sample (underlined), and both (**bold**).

Most associated marker	Chr.	Position (cM)	Whole collection		*Durum* sub-sample		Other associated markers in the region	Chr. interval	Literature
			P (Q+K)	R^2^ (%)	P (Q+K)	R^2^ (%)			
wPt7339	1A-3	24.9	3.18E-02	2	-	-	-	-	-
wPt0308*	1B	0.0	-	-	*6.79E-03*	*7*	-	-	-
***Xcfd15***	1B	14.1	3.03E-02	2	*3.29E-02*	*4*	-	-	-
***wPt4726***	1B	40.0	2.01E-02	2	*3.75E-02*	*4*	-	-	[32]
wPt5907	1B	167.5	1.52E-02	2	-	-	-	-	[32]
wPt4651	1B	215.6	3.24E-02	2	-	-	wPt5577, wPt1770, wPt8245	0.9	-
*wPt5788*	2B	69.3	-	-	*2.35E-02*	*4*	*wPt5513*	0.2	[68]
Barc45	3A-2	38.1	3.64E-02	2	-	-	-	-	-
***Xgwm1042***	3A-2	68.8	3.81E-02	2	*2.52E-02*	*4*	-	-	[32]
wPt4398*	3A-2	146.4	-	-	*4.19E-02*	*3*	-	-	-
***Xgwm937***	4A	46.3	1.11E-04	6	*9.32E-04*	*9*	-	-	[32]
wPt4660	4A	53.0	1.57E-02	2	-	-	-	-	[32]
***wPt7939***	4A	94.0	3.20E-02	2	*3.82E-02*	*4*	wPt5857, wPt3810	2.1	-
wPt4596	4A	109.0	1.60E-02	2	-	-	-	-	-
***wPt6757***	4A	128.5	1.76E-02	2	*2.24E-02*	*4*	wPt5055, *wPt1007*, wPt3729*	5.9	-
***Xgwm495***	4B-2	84.0	8.45E-03	3	*1.01E-02*	*5*	-	-	[52]
Xgwm154	5A-1	24.3	1.05E-02	3	-	-	-	-	-
wPt5588	5A-1	78.3	1.28E-02	3	-	-	-	-	-
***wPt7201***	5A-1	120.4	2.15E-02	2	*2.78E-02*	*4*	-	-	-
*wPt5135*	5A-2	50.1	-	-	*3.73E-02*	*3*	*wPt0152*	0.2	[52]
***wPt3661***	5B-1	111.4	1.02E-02	3	*4.57E-02*	*4*	-	-	[8]
wPt2305	7B	138.3	1.79E-02	2	-	-	-	-	-
*wPt8312*	7B	151.3	-	-	*2.03E-02*	*4*	*wPt0841*, *wPt9133*,	0.2	[52]
***wPt5138***	7B	244.6	4.36E-02	2	*9.50E-03*	*6*	-	-	[32]
wPt9746*	7B	258.9	-	-	*1.59E-02*	*5*	wPt6276	0.1	-

Asterisks (*) indicate DArT markers putatively under selection.

**Table 8:** Representative markers significantly associated with the heading date trait, for the whole collection (normal), the *durum* sub-sample (underlined), and both (**bold**).

Most associated marker	Chr.	Position (cM)	Whole collection		*Durum* sub-sample		Other associated markers in the region	Chr. interval	Literature
			P (Q+K)	R^2^ (%)	P (Q+K)	R^2^ (%)			
wPt4735*	1A-1	4.2	2.76E-02	2	-	-	-	-	-
wPt6280	1A-1	20.2	4.03E-02	2	-	-	-	-	[33]
Xcfd15	1B	14.1	4.16E-02	2	-	-	-	-	-
wPt3103	1B	51.5	3.75E-02	2	-	-	wPt6012*	0.8	[33]
*wPt0459*	1B	141.6	-	-	*4.46E-02*	*3*	-	-	[33]
wPt4533	2A-1	21.2	3.83E-02	2	-	-	-	-	[32]
*wPt2600*	2B	77.2	-	-	*2.89E-02*	*4*	*wPt7320*	0.2	[33]
wPt1140*	2B	158.3	3.97E-02	2	-	-	-	-	[32]
wPt2724	2B	175.0	1.07E-03	5	-	-	-	-	[33]
Barc45	3A-2	38.1	2.57E-02	2	-	-	-	-	-
***Xgwm1042***	3A-2	68.8	2.94E-03	4	*1.67E-03*	*8*	-	*-*	[32]
***wPt1562***	3A-2	125.1	3.95E-02	2	*2.58E-02*	*4*	*wPt3816*, *wPt2659*	0.2	[33]
*wPt9160*	3A-2	171.2	-	-	*2.85E-02*	*4*	-	-	[33]
*wPt1516*	3B-1	9.1	-	-	*4.04E-02*	*3*	-	-	-
*wPt8079*	3B-1	14.4	-	-	*2.00E-02*	*4*	*wPt3921*	0.0	-
***wPt3536***	3B-1	24.9	2.64E-02	2	*2.58E-02*	*5*	wPt9410, wPt5064, wPt0302*	2.1	[33]
*wPt9088*	3B-1	60.4	-	-	*4.65E-02*	*3*	wPt6216, wPt6239	4.0	[33]
wPt2280*	3B-1	168.2	-	-	*9.40E-03*	*6*	-	-	-
wPt9488	3B-2	17.2	3.29E-02	2	-	-	-	-	-
***Xgwm937***	4A	46.3	3.11E-02	2	*7.41E-03*	*6*	-	*-*	[32]
***wPt1007***	4A	125.8	2.30E-03	4	*3.00E-02*	*4*	*wPt9196*, *wPt3449*, wPt3729*	6.6	[33]
wPt7167	5B-1	96.2	3.69E-02	2	-	-	-	-	-
*rPt7889*	5B-1	153.5	-	-	*1.16E-02*	*5*	-	-	-
*rPt3114*	5B-2	7.8	-	-	*4.09E-03*	*7*	-	-	-
wPt9690	6A	48.5	3.82E-02	2	-	-	wPt2573	0.2	-
***wPt1375***	6A	159.3	3.25E-02	2	*2.77E-02*	*4*	**wPt5572**	0.2	[32]
Xgwm193	6B	107.8	2.80E-02	2	-	-	-	-	-
wPt3883	7A-2	84.7	1.57E-02	3	-	-	wPt7734, wPt9796	0.5	[53]
wPt4877	7A-3	41.7	4.95E-02	2	-	-	-	-	[33]
wPt5940	7A-3	137.3	2.50E-02	2	-	-	-	-	-

Asterisks (*) indicate DArT markers putatively under selection.

**Table 9:** Representative markers significantly associated with the protein content trait, for the whole collection (normal), the *durum* sub-sample (underlined), and both (**bold**).

Most associated marker	Chr.	Position (cM)	Whole collection		*Durum* sub-sample		Other associated markers in the region	Chr. interval	Literature
			P (Q+K)	R^2^ (%)	P (Q+K)	R^2^ (%)			
wPt4676*	1A-1	31.4	3.85E-02	2	-	-	-	-	[10]
*wPt2706*	1B	37.4	-	-	*1.42E-02*	*5*	*wPt5562*, wPt5899*	0.0	-
wPt3103	1B	51.5	3.70E-03	4	-	-	-	-	[73]
wPt9409	1B	130.2	4.98E-02	2	-	-	wPt1247	0.0	-
*wPt9028*	1B	204.9	-	-	*4.72E-03*	*6*	wPt8866*	0.4	-
***wPt7626***	2A-1	38.5	1.94E-02	2	*3.71E-02*	*4*	-	-	[9]
wPt8826	2A-1	146.4	4.59E-03	4	-	-	-	-	-
Xgwm311	2A-3	9.3	1.93E-02	2	-	-	-	-	-
***BQ170801***	2B	10.6	4.37E-03	4	*2.10E-03*	*8*	-	*-*	*-*
*wPt2600*	2B	77.2	-	-	*2.97E-02*	*4*	*wPt7320*	0.2	-
*wPt5736*	2B	164.7	-	-	*3.14E-02*	*4*	wPt8284*, wPt4917*	0.4	-
***wPt2724***	2B	175.0	4.14E-02	2	*3.40E-03*	*7*	wPt0906, rPt6122*	3.1	-
***Xgwm1042***	3A-2	68.8	1.63E-02	3	*8.80E-03*	*5*	-	*-*	[10]
*wPt3816*	3A-2	125.0	-	-	*3.23E-02*	*4*	*wPt1562*	0.1	-
wPt9160	3A-2	171.2	2.47E-02	2	-	-	wPt0398	2.8	-
wPt0142*	3A-3	11.0	-	-	*1.67E-02*	*5*	-	-	-
wPt2280*	3B-1	168.2	-	-	*1.09E-02*	*5*	-	-	-
*Xgwm299*	3B-2	28.1	-	-	*4.73E-02*	*3*	-	-	-
*wPt0990*	3B-2	42.2	-	-	*2.55E-02*	*4*	wPt6000*	0.3	-
wPt4660	4A	53.0	3.02E-02	2	-	-	-	-	-
*wPt7939*	4A	94.0	-	-	*1.12E-02*	*5*	wPt6728	3.1	-
wPt5112	4A	123.3	4.25E-03	4	-	-	wPt9196, wPt0610*	4.3	-
Xgwm495	4B-2	84.0	3.99E-02	2	-	-	-	-	-
*Xgwm1084*	4B-2	106.9	-	-	*8.56E-03*	*5*	-	-	-
Xgwm154	5A-1	24.3	4.03E-02	2	-	-	-	-	-
***Xgwm865***	5A-2	0.0	1.24E-03	5	*4.13E-04*	*10*	-	*-*	[10]
*Xwmc235*	5B-1	175.3	-	-	*1.51E-02*	*5*	-	-	-
wPt7486	6A	50.8	1.79E-02	3	-	-	-	-	-
wPt8944	6A	162.1	5.66E-03	3	-	-	wPt4229, wPt4445, wPt9474, wPt6829*	7.1	[8]
wPt2632*	6A	173.0	1.83E-02	3	-	-	-	-	-
wPt8721	6B	89.9	9.22E-03	3	-	-	-	-	[51]
wPt6184	7A-1	2.5	2.97E-02	2	-	-	-	-	-
wPt3883	7A-2	84.7	4.27E-02	2	-	-	-	-	-
wPt6842	7A-3	24.3	4.33E-03	4	-	-	-	-	-
Xgwm1017	7A-3	82.0	1.51E-02	3	-	-	-	-	-
wPt1976*	7A-3	122.9	4.70E-02	2	-	-	-	-	-
*Xwmc606*	7B	37.5	-	-	*2.84E-02*	*4*	-	-	-
*Xgwm537*	7B	75.9	-	-	*8.65E-03*	*5*	-	-	-
***wPt2305***	7B	138.3	1.91E-02	3	*3.31E-02*	*4*	-	*-*	[10]

Asterisks (*) indicate DArT markers putatively under selection.

**Table 10:** Representative markers significantly associated with the thousand kernel weight trait, for the whole collection (normal), the *durum* sub-sample (underlined), and both (**bold**).

Most associated marker	Chr.	Position (cM)	Whole collection		*Durum* sub-sample		Other associated markers in the region	Chr. interval	Literature
			P (Q+K)	R^2^ (%)	P (Q+K)	R^2^ (%)			
wPt6280	1A-1	20.2	1.26E-02	3	-	-	-	-	-
*wPt2014*	1A-2	70.5	-	-	*4.65E-02*	*3*	-	-	-
Xgwm124	1B	143.2	4.77E-03	4	-	-	-	-	[32]
*wPt4361*	1B	208.3	-	-	*5.26E-03*	*6*	-	-	-
*wPt4533*	2A-1	21.2	-	-	*1.12E-02*	*5*	*wPt6245*, tPt1041*	4.6	[32]
wPt3037	2A-1	97.9	3.01E-02	2	-	-	wPt2435	2.1	-
*wPt9277*	2A-3	16.0	-	-	*2.88E-02*	*4*	*wPt9793*	0.4	[32]
***wPt1064***	2B	88.9	1.65E-02	3	*6.94E-03*	*6*	***wPt0615***	0.1	[32]
wPt8284*	2B	164.9	7.86E-03	3	-	-	wPt5736	0.2	[32]
***wPt7506***	2B	223.5	3.76E-02	2	*1.09E-02*	*5*	wPt8776	5.0	[55]
*Xgwm1042*	3A-2	68.8	-	-	*4.05E-02*	*3*	-	-	[32]
*tPt1002*	3A-2	120.3	-	-	*2.84E-03*	*7*	-	-	-
tPt7492*	3A-2	126.7	-	-	*1.73E-03*	*8*	*wPt5125*, *wPt5133*	0.0	-
wPt1888*	3A-2	157.1	3.29E-02	2	*1.62E-02*	*4*	***wPt3978***	0.1	-
wPt2144*	3A-2	171.1	-	-	*2.93E-03*	*7*	*wPt4545*, *wPt9160*, *wPt0398*	5.6	-
wPt6785	3B-1	196.5	3.57E-02	2	-	-	-	-	-
*wPt8959*	3B-2	23.6	-	-	*9.02E-03*	*5*	*wPt3480*, wPt3342*	0.3	-
Xgwm937	4A	46.3	3.63E-02	2	-	-	-	-	-
wPt6502	4A	119.8	1.79E-02	3	-	-	wPt7821	0.0	[73]
Xgwm1084	4B-2	106.9	1.85E-02	2	-	-	-	-	[32]
***wPt5588***	5A-1	78.3	4.59E-02	2	*2.97E-03*	*7*	-	*-*	*-*
*wPt7201*	5A-1	120.4	-	-	*2.45E-02*	*4*	-	-	[56]
Xgwm865	5A-2	0.0	4.09E-03	4	-	-	-	-	-
*wPt0498*	5B-1	121.0	-	-	*1.31E-02*	*5*	-	-	[73]
*rPt7889*	5B-1	153.5	-	-	*1.72E-03*	*8*	-	-	-
*rPt3114*	5B-2	7.8	-	-	*4.73E-03*	*6*	-	-	-
*wPt8920*	5B-3	4.8	-	-	*4.81E-02*	*3*	wPt7665*	4.8	-
wPt9832	6A	24.6	4.52E-02	2	-	-	-	-	-
wPt7663	6A	41.0	2.28E-02	2	-	-	-	-	-
*wPt5834*	6A	70.3	-	-	*8.99E-03*	*5*	-	-	-
wPt2632*	6A	173.0	-	-	*1.23E-02*	*5*	*wPt4229*	9.8	[32]
wPt3116	6B	17.5	1.47E-02	3	-	-	-	-	[32]
***wPt4742***	6B	61.4	2.30E-02	2	*8.91E-03*	*5*	wPt2297, wPt0470	1.6	-
wPt8194	6B	76.6	1.39E-02	3	-	-	-	-	[56]
*wPt2564*	6B	108.9	-	-	*1.94E-02*	*5*	-	-	[32]
*wPt5092*	7A-1	3.9	-	-	*1.59E-03*	*8*	*tPt9948*, *wPt6184*, wPt3572	7.5	[32]
*wPt9314*	7A-2	92.8	-	-	*1.42E-02*	*5*	-	-	-
Xgwm1017	7A-3	82.0	2.24E-02	2	-	-	-	-	[56]
Xwmc606	7B	37.5	3.36E-02	2	-	-	-	-	[56]
*Xgwm537*	7B	75.9	-	-	*7.40E-03*	*5*	-	-	[32]
wPt1715	7B	203.4	4.69E-02	2	-	-	-	-	[32]
wPt2449	7B	248.4	4.92E-02	2	-	-	-	-	-

Asterisks (*) indicate DArT markers putatively under selection.

Altogether, in the whole collection, 221 MTAs (Table 6) were identified for 189 loci (16 SSRs and 173 DArTs), of which 41.3% (78 loci) are unmapped on the durum wheat consensus map. From this number of markers, 160 (84.7%) are specific for a single trait (PH, 28; HD, 43; PC, 44; TKW, 45 loci), and the rest (29 loci) consist of associations of up to three traits. HD and PC are involved in the highest number of MTAs (61, 60, respectively), followed by TKW (55) and PH (45) (Table S2).

In the *durum* sub-sample, 191 MTAs (Table 6) were identified for 156 loci (14 SSRs and 142 DArTs), of which 39.7% (62 markers) are unmapped, and 80.8% (126 loci) are specific for a single trait (PH, 26; HD, 21; PC, 31; TKW, 48 loci). The Xgwm1042 SSR is the only locus that identified MTAs for four traits, the rest (29 loci) consist of associations of up to three traits. The lowest numbers of MTAs are associated with PH and HD (41, 42, respectively), followed by PC (50), with the maximum for TKW (58) (Table S2).

There were 51 loci (11 SSRs and 40 DArTs) identified as MTAs for the same trait in both the whole collection and the *durum* sub-sample, of which 30 map on the durum wheat consensus map. Of these 51 MTAs, 47.1% (24 loci) are specific for a single trait (PH, 5; HD, 6; PC, 5; TKW, 8 loci), while 27 are multi-trait MTAs. Overall, PH is involved in the highest number of multi-trait MTAs (15) identified in the two samples, followed by HD (13), TKW (12) and PC (11).

### Mapped MTAs

There were 128 mapped MTAs (Table S2; Figures 2 and 3) in the whole collection (corresponding to 111 loci) and 110 in the *durum* sub-sample (corresponding to 94 loci). For the whole collection, out of the 111 loci (14 SSRs and 97 DArTs) for which there is at least one MTA, 86.5% (96 loci) are associated with only one trait, while the rest consist of associations of up to three traits (15 loci). In the *durum* sub-sample, out of the 94 loci (11 SSRs and 83 DArTs), 85.1% (80 loci) are single-trait MTAs. Among these 111 (whole collection) and 94 (*durum* sub-sample) loci, 16 and 15, respectively, are putatively under selection.

The MTAs are located in all of the chromosomes of both genome A and B. The highest number of MTAs identified in the two samples are on chromosome 3A (32 MTAs for 17 loci), followed by chromosome 4A (30 MTAs for 18 loci) and chromosome 2B (27 MTAs for 17 loci), with the lowest on chromosome 1A (6 MTAs for 5 loci) and chromosome 4B (5 MTAs for 2 loci). Considering the homoeologous groups, group 3 contains the highest number of MTAs (53 for 36 loci), followed by group 2 with 38 MTAs (27 loci); with the lowest numbers for groups 5 (25 MTAs) and 6 (27 MTAs), for 14 and 22 loci, respectively. Altogether, the A-genome has a higher number of MTAs (127), for 83 loci, compared to the B-genome with 122 MTAs, for 84 loci.

The MTAs that are identified for each trait are presented in Tables 7, 8, 9 and 10. Concurrence between our association mapping results in tetraploid wheat and those reported in previous wheat association mapping and linkage mapping studies was observed in several cases. To compare our data with data obtained in other studies, we considered the most recently published information on genes and QTL mapping for these traits (PH, HD, PC, TKW) and for the domestication syndrome, which includes genes for photoperiod insensitivity (*Ppd*), vernalisation (*Vrn*), earliness *per se* (*Eps*), dwarfism (*Rht*), grain protein content (*Gpc*), and domestication syndrome traits (*Tg*, *Br*, *sog*, *Q* locus).

For PH, 26 (whole collection) and 22 (*durum* sub-sample) MTAs were identified (Table 7). Overall, these MTAs (which correspond to 38 loci) detected 25 putative QTL regions and are located on 9 different chromosomes: 1A, 4B, 5B (1 marker each), 2B (2 markers), 3A (3 markers), 5A (5 markers), 7B (7 markers), 1B (8 markers) and 4A (10 markers). The phenotypic variation of each of these loci ranged from 2% (13 loci) to 9% (1 locus). Overall, 10 loci are confirmed as MTAs for PH in both the whole collection and the *durum* sub-sample, and 4 loci are putatively under selection [43]. As expected, the PH-related QTL on chromosome 4B that was tagged by Xgwm495 (84.0 cM), which has a phenotypic variation of 3% (whole collection) to 5% (*durum* sub-sample), was identified in both the whole collection and the *durum* sub-sample in the genomic region harbouring the growth habit gene *Rht-B1* [33]. At about 5 cM from this MTA, Zhang et al. [52] reported a PH-related QTL associated with the Xgwm513 (78.8 cM) SSR locus. Three QTLs that show relatively high *R^2^* values (from 6% to 9%) are located on chromosome arms 1BS, 4AL and 7BL, and are tagged by the markers wPt-0308, Xgwm937 and wPt-5138, respectively (Table 7; Figures 2 and 3).

The first putative QTL was identified by the DArT marker wPt-0308, which is also putatively under selection [43], and is about 14 cM from another PH-related QTL, tagged by the Xcfd15 SSR marker, associated in both the whole collection (*R^2^*, 2%) and the *durum* sub-sample (*R^2^*, 4%). The Xcfd15 marker is also associated to multi-trait MTAs for PH and HD. In this chromosome region, Maccaferri et al. [32] identified QTLs for yield components (TKW and kernel number spike [KNS]) using association mapping on a panel of 189 durum wheat varieties.

The second putative QTL is associated to the Xgwm937 SSR marker, and was identified in both the whole collection and the *durum* sub-sample, with *R^2^* of 6% and 9%, respectively. This marker is also associated to a multi-trait MTA for PH, HD and TKW. At 7 cM from this MTA, the wPt-4660 locus is associated with PH in the whole collection, and in the same region, Maccaferri et al. [32] reported the presence of QTLs for PH, and also for grain yield and HD.

The third QTL is in the distal region on chromosome 7BL and was identified by the wPt-5138 (244.6 cM) marker, associated in both the whole collection (*R^2^*, 2%) and the *durum* sub-sample (*R^2^*, 6%). In the same region, the wPt-9746 (258.9 cM) marker is also putatively under selection, and is associated to a MTA only in the *durum* sub-sample (*R^2^*, 5%). In the same chromosome region, three MTAs (wPt-8312, wPt-0841, wPt-9133) located at 151 cM that span an interval map of 0.2 cM are associated with PH only in the *durum* sub-sample (*R^2^*, 4%, for each MTA). In a panel of 154 common wheat accessions, Zhang et al. [52] used association mapping analysis to identify a PH-related QTL at the Xgwm302 SSR locus mapped in the durum wheat consensus map [44], at about 4 cM from the MTAs in the present study.

The HD is associated to 31 (whole collection) and 23 (*durum* sub-sample) MTAs. These MTAs (corresponding to 47 loci) detected 30 putative QTL regions (Table 8; Figures 2 and 3) and are located on 11 different chromosomes (not including chromosomes 4B, 5A and 7B). The phenotypic variation of each of these loci ranges from 2% (20 loci) to 8% (1 locus). Overall, 7 loci are confirmed as MTAs for HD in both the whole collection and the *durum* sub-sample, and 6 markers are putatively under selection. As expected, the two homoeologous copies of *Ppd-1* on the chromosomes of group 2 were close to the MTAs identified for HD in our association mapping panel. In particular, in the region on chromosome arm 2AS, the wPt-4533 (21.2 cM) locus was identified as a MTA for HD only in the whole collection. This MTA is close on the interval map, among the Xgwm359 (38.9 cM) and Xgwm122 (25.4 cM) markers, within which Griffiths et al. [53] mapped the photoperiod insensitivity locus (*Ppd*-A1) using meta-QTL analysis. Then on chromosome arm 2BS, Le Gouis et al. [33] mapped the *Ppd-B1* locus close to the wPt-6932 marker, located at 66.5 cM on the durum wheat consensus map. In the present study, at a distance of ∼10 cM from the wPt-6932 marker, one HD-related QTL was detected from the wPt-2600 and wPt-3720 markers, only in the *durum* sub-sample, with *R^2^* for both of 4% (Table 8; Figure 3). Considering the vernalisation requirement loci (*Vrn1*, *Vrn2*, *Vrn3*), in the present study, there are HD-related QTLs only in the chromosome regions harbouring the *Vrn-B1* and *Vrn-A3* loci, which are located on chromosome arms 5BL and 7AL, respectively, as reported by Le Gouis et al. [33]. In particular, the HD-related QTL associated with the rPt-7889 (153.5 cM) locus on chromosome arm 5BL, with *R^2^* of 5%, is close to the *Vrn-B1* locus. Then, on chromosome arm 7AL and very near to the *Vrn-A3* locus, there are three MTAs (wPt-3883, wPt-7734, wPt-9796) associated to HD only in the whole collection, with a maximum *R^2^* of 3%.

On chromosome arm 3BS in an interval map of ∼16 cM, 7 loci were identified as MTAs for three HD-related QTLs (Table 8; Figure 3), with *R^2^* from 2% to 5%. The first two of these QTLs are only in the *durum* sub-sample, while the third QTL was identified from 4 MTAs, of which the wPt-3536 (24.9 cM) locus is associated to HD in both the whole collection (*R^2^*, 2%) and the *durum* sub-sample (*R^2^*, 5%), and the wPt-0302 locus is putatively under selection. The wPt-0302 marker was associated with components of earliness in an association mapping study conducted by Le Gouis et al. [33], and in the same region Maccaferri et al. [32] identified QTLs for HD, PH and KNS. On chromosome arm 3AL, the Xgwm1042 (68.8 cM) SSR marker identified a MTA for HD in both the whole collection (*R^2^* of 4%) and the *durum* sub-sample (*R^2^*, 8%), which is a multi-trait MTA associated to the other traits considered in the present study. Close to this MTA, Blanco et al. [10] reported a QTL for PC and TKW, using a recombinant inbred line population derived from two durum wheat cultivars (Svevo, Ciccio), and Maccaferri et al. [32] used association mapping analysis to identify putative QTLs for PH, HD and TKW. Overall, all of the 10 HD-related QTLs (from 18 MTAs) on the group 3 chromosomes that were identified in the present study (Table 8; Figures 2 and 3) have also been confirmed in two other studies that were based on association and linkage QTL mapping [32]–[33].

In the distal region on chromosome arm 4AL in an interval map of 6.6 cM, the present study identified 4 MTAs, each of which explain 4% of the phenotypic variation, where the wPt-1007 DArT locus was detected in both the whole collection and the *durum* sub-sample (Table 8; Figure 2). In this same chromosome region, the studies of Maccaferri et al. [32] and Le Gouis et al. [33] showed HD-related QTLs. Finally, there are two MTAs (wPt-1375, wPt-5572) mapped on chromosome arm 6AL (159 cM) identified in both the whole collection and the *durum* sub-sample (*R^2^* from 2% to 4%). The putative QTL detected in this chromosome region is close to markers identified for significant associations reported by Maccaferri et al. [32].

The PC is involved in 38 (whole collection) and 26 (*durum* sub-sample) MTAs. These MTAs (corresponding to 58 loci) detected 39 putative QTL regions (Table 9; Figures 2 and 3) spread across all of the chromosomes. The phenotypic variation associated with these loci ranges from 2% (20 loci) to 10% (1 locus). Overall, there are 6 MTAs for PC identified in both the whole collection and the *durum* sub-sample, and 13 loci are putatively under selection. As expected, on chromosome arm 6BS, and tagged by the wPt-8721 (89.9 cM) DArT MTA (*R^2^*, 3%), the PC-related QTL was identified in the genomic region harbouring the *Gpc-B1* gene, among the Nor2 (77.3 cM) and Xgwm193 (107.8 cM) markers, as reported by Uauy et al. [54].

On chromosome 2B, four putative QTLs that show high *R^2^* were identified by the BQ170801, wPt-2600, wPt-5736 and wPt-2724 MTAs (Table 9; Figure 3). The first QTL was detected in both the whole collection and the *durum* sub-sample (*R^2^* from 4% to 8%) on chromosome arm 2BS. In the same chromosome region, the second PC-related QTL was identified by two multi-trait MTAs (wPt-2600, wPt-7320) and is associated only in the *durum* sub-sample, with *R^2^* of 4%. The other two PC-related QTLs were identified on chromosome arm 2BL in an interval map of ∼10 cM, and were detected from 6 MTAs (*R^2^* from 2% to 7%), of which three were identified as putatively under selection (Table 9; Figure 3). The putative QTL detected in this chromosome region is close to markers identified as significant associations for PH, HD, TKW and KNS reported by Maccaferri et al. [32].

On chromosome arm 5AL, the Xgwm865 marker identified in both the whole collection and the *durum* sub-sample with *R^2^* from 5% to 10% is a multi-trait MTA associated also with TKW. In the same chromosome region, Blanco et al. [10] reported a QTL for grain protein content and KNS.

In an interval map of ∼10 cM on chromosome arm 6AL, two PC-related QTLs were identified from 6 MTAs only in the whole collection (*R^2^* from 2% to 3%), for which two DArT markers (wPt-2632, wPt-6829) are putatively under selection. In the same chromosome region, and using linkage mapping analysis for kernel characteristics in a segregating population of common wheat, Tsilo et al. [8] reported a QTL for grain protein content.

The TKW is associated with 33 (whole collection) and 39 (*durum* sub-sample) MTAs. These MTAs (corresponding to 65 loci) detected 42 putative QTL regions (Table 10; Figures 2 and 3) distributed across all of the chromosomes. The phenotypic variation of each of these loci ranges from 2% (15 loci) to 8% (5 loci). Overall, 7 loci were confirmed as MTAs for TKW in both the whole collection and the *durum* sub-sample, and 8 loci are putatively under selection. There are three TKW-related QTLs on chromosome 2B; the first was identified on chromosome arm 2BS from two MTAs (wPt-1064, wPt-0615) and was detected for the whole collection and in the *durum* sub-sample, with *R^2^* from 3% to 6% (Table 10; Figure 3). The other two QTLs located on chromosome arm 2BL were identified by two MTAs for each QTL, for which the wPt-8284 marker is putatively under selection and wPt-7506 is associated in the whole collection (*R^2^*, 2%) and in the *durum* sub-sample (*R^2^*, 5%) (Table 10; Figure 3). The putative QTLs identified on chromosome 2B are close to markers that were identified as significant associations in the studies of Maccaferri et al. [32], Blanco et al. [10] and Yu et al. [55], for grain yield and yield components (TKW and KNS).

On chromosome arm 3AL, five putative TKW-related QTLs (*R^2^* from 3% to 8%) were identified, of which four are detected only in the *durum* sub-sample dataset. In particular, in the distal region of this chromosome arm, there are 3 putative QTLs identified by 9 MTAs, of which three markers (tPt-7492, wPt-1888, wPt-2144) are putatively under selection, and two (wPt-9160, wPt-0398) are multi-trait MTAs associated also to PC and HD (Table 10; Figure 2). The QTL identified by two MTAs for wPt-1888 and wPt-3978 is the only one detected in both the whole collection and the *durum* sub-sample (*R^2^* from 2% to 4%). The putative QTLs detected in this chromosome region are close to markers identified as having significant associations in others studies of QTL mapping, as reported by Maccaferri et al. [32] and Peleg et al. [56].

### Unmapped MTAs

Three SSRs and 378 DArT markers were not located on the durum wheat consensus map. The three SSR loci (Xgwm408, Xgwm1066, Xgwm1093) were identified as multi-trait MTAs, and the first two were detected in both the whole collection and the *durum* sub-sample, while the third marker was detected only in the *durum* sub-sample. Out of the 378 DArT markers, 55 were identified as MTAs only in the whole collection (12 putatively under selection), 38 loci were detected as MTAs only in the *durum* sub-sample (13 putatively under selection), and 21 are MTAs in both samples (5 putatively under selection).

Linkage disequilibrium analysis was performed between 114 unmapped DArTs identified as MTAs (in the whole collection and/or in the *durum* sub-sample) and 592 DArT loci mapped on the durum wheat consensus map. For 11 of these unmapped MTAs that are associated with all of the traits except PH, strong or complete LD was detected for the mapped markers, and therefore they are considered to be mapped in the same region. Five and 6 MTAs are located on genomes A and B, respectively, and are trait-specific MTAs, except for the wPT-9577 locus on chromosome arm 3BL, which is associated with PC and TKW.

With few exceptions, all of the unmapped markers that are located on the consensus map using LD confirm the positions of MTAs identified using mapped markers. There are new MTAs associated to unmapped markers in only two cases:

1. wPt-8140 MTA associated only in the *durum* sub-sample is associated to TKW, while the closely linked wPt-6216 locus, on chromosome 3B, is associated with HD (Table 9; Figure 3);
2. wPt-9577 confirms the TKW MTA on chromosome 3BL, tagged by the closely linked wPt-6785 locus (Table 10; Figure 3), which is also a MTA for PC.

## Discussion

In the present study, we have reported the results of a map-based analysis of the genetic diversity, population structure, and LD patterns of a panel of tetraploid wheat accessions (230 inbred lines) that is constituted by a large set of durum wheat varieties and by a representative sample of *T. turgidum* evolutionary lineages, including wild and domesticated accessions. Using this structured panel, we conducted a parallel analysis of marker-trait association for four key agronomic traits on two samples with different LD levels and structures, with the possibility of cross validation of the association mapping results and their comparison with outlier analysis conducted in a previous study [43].

The analysis of the literature on QTL mapping for the same four traits confirms many of our MTAs and clearly indicates that our approach was successful for the detection of relevant QTLs for the traits investigated. Most likely, these positive results are also associated with the high heritability observed for the target traits. Considering the whole collection, the heritabilities for all of the traits exceeded 0.85 (except for PC at 0.67). Indeed, association mapping is strongly influenced by the heritability and by the quality of the phenotypic data [24], [57].

Our study was greatly aided by the availability of the consensus map that was reported in Marone et al. [44], which is a very useful tool to compare the locations of the markers (SSRs and DArTs) from the present study with other markers reported in other studies. Our data rely on a dataset that for both marker number and sample size (∼1000, including SSR and DArT markers) is larger than that of similar association mapping studies that have been carried out in both durum and bread wheat [22], [25], [30], [32], [33], [52], [58], [59].

### Genetic diversity and linkage disequilibrium

We were able to analyse the genetic diversity of our samples using mapped markers that are well distributed among the chromosomes, with a density of 1 marker every 4.0 cM. Our data indicate that the level of diversity among the chromosomes is heterogeneous in the *durum* sub-sample compared with that observed in the whole collection (where many naked and hulled tetraploid types were included, along with a few wild emmer genotypes), with a relatively strong reduction in diversity in the *durum* sub-sample for chromosome 1A. The genetic determinants of PC and TKW have been mapped previously for chromosome 1A [60]–[61]. The selection in durum wheat due to domestication, and particularly to obtain the modern varieties, has been targeted to meet very strict agronomic and quality (high PC) requirements [43], so for chromosome 1A, our data can be explained by the occurrence of a higher loss of diversity in the regions harbouring loci involved in PC and TKW.

The durum wheat consensus map developed by Marone et al. [44] was needed for the present study, to determine the loci positions for the combination of the results of different segregation analysis into a single map. Although consensus maps inherently contain errors in marker order and linkage distance, the pattern of LD detected was in good agreement with the consensus marker positions. Thus we can be confident in our analysis of the LD pattern and decay over genetic distances.

The means of *r^2^* of all of the interchromosomal pairs in the whole collection and in the *durum* sub-sample are 0.023 and 0.029, respectively, which are slightly higher than the 0.022 described by Breseghello and Sorrells [47] and 0.019 reported by Neumann et al. [25] in populations of smaller sizes to that investigated here, and with a similar number of marker pairs. This indicates that the LD due to the population structure is very similar between the durum wheat, bread wheat and barley accessions studied here and in the cited literature.

For the intrachromosomal LD, in each dataset the percentage of locus pairs showing significant LD is highest for adjacent locus pairs (<10 cM), lower for locus pairs on the same chromosome (>10 cM), and lowest for locus pairs on independent chromosomes. In the *durum* sub-sample, the LD decay (the point in which the LOESS curve intercepts the critical *r^2^*) was at *ca.*18 cM, while in the whole collection the decay was more rapid, at 14 cM. The level of LD in our *durum* sub-sample was larger than that observed in durum wheat by Maccaferri et al. [32], [39], and in common wheat by Breseghello and Sorrells [47], Chao et al. [58] and Somers et al. [22]. The *durum* sub-sample and the Q1 group follow the same pattern in the decay of the LD, whereas in the Q2 group, the LD falls at a distance of 5 cM. Overall, the level of LD was greater in the *durum* sub-sample compared to the Q2 group, which includes wild and domesticated accessions.

Overall, this picture indicates that in tetraploid wheat the pattern of LD is extremely population dependent and related to the process of domestication. This has also been reported in other autogamous species, such as barley, where the genome-wide LD extended from 10 cM to 15 cM [62]–[65]. The higher level of LD found in the *durum* sub-sample compared to the wild and domesticated accessions (the Q2 group) can be explained by the different levels of historical recombination in the two samples, and because of the effects of the bottlenecks of domestication and breeding, as increases in the level of LD. Our data can be explained under just a neutral model, where the effective recombination rate due to the effective population size along with the physical and recombination distance between the loci is the major determinants of the pattern of LD. For this reason, using the LD pattern [65], we were also able to map the loci that were unmapped in the consensus map [44], and in particular those that were MTAs.

However, we cannot exclude the role of selection in favouring the conservation of haplotype blocks, along with the possible role of inversions and other chromosomal mutations. On the other hand, we do not observe much evidence of such phenomena, and only in a few cases did we observe evidence of long distance or interchromosomal LD, as has been reported for other species where epistatic selection might have been the cause of the observed patterns [30], [66]. Only in one case did we observe complete LD at long-range distance, on chromosome 3A, in which the wPt-4545 marker is in complete LD with wPt-3978 and wPt-1888 at 11.3 cM in all of the datasets used in the present study. In other cases, on chromosome 2A there is the wPt-8925 marker in high LD (*r^2^*>0.75) with three co-mapping DArT markers (wPt-3611, wPt-0277, wPt-3744) at 18.3 cM in all of the datasets, with the exception of the Q2 group. In the *durum* sub-sample, we observe other cases of long-range LD between loosely linked markers on chromosomes 6A and 3B (in particular, for the linkage group 3B-2) at a distance of ∼14 cM.

However, in some cases, the pattern of LD might be indicative of areas of the genome where selection and chromosomal mutations might have reduced the effective recombination rate (*r_e_*). Indeed, the LD decay in the whole collection is different for the single chromosomes. For some of the chromosomes, the LD decay is in the same range as for the whole collection, with the exceptions of chromosomes 4A and 3B (in particular, for the linkage group 3B-2), in which the decay is at 5 cM and 8 cM, respectively, and of chromosomes 1B and 6A, with the decay at 18 cM and 20 cM.

### Genome-wide association mapping

Compared to the MLM, the GLM identifies a greater number of MTAs. However, the GLM has a high risk of false-positive detection. Indeed, with the GLM, too many associations appear that are not detected with the MLM, while most of the MLM MTAs are confirmed by the GLM. For this reason, in the present study, only the MTAs identified with the MLM are considered [38], [67].

Overall, our data provide insight into the genetic architecture of important agronomic traits for durum wheat (PH, HD, PC, TKW). In total, we identified 89 QTLs for these traits, from 25 QTLs for PH, to 42 QTLs for TKW. Some genomic regions harbour QTLs for more than one trait, and based on the map comparisons, 44 QTLs concur with previously mapped QTLs in various studies of association mapping and linkage mapping in durum and common wheat, and with genes for photoperiod insensitivity (*Ppd*), vernalisation (*Vrn*), earliness *per se* (*Eps*), dwarfism (*Rht*), and grain protein content (*Gpc*). By comparing our data with several other studies in wheat, we were able to confirm the positions of many major genes and QTLs for the considered traits, while obtaining a validation of our results for the novel QTLs that were detected.

Considering PH, and in particular the *Rht-B1* genes located on chromosome 4B and in the homeologous group 2 [52], we observed that for chromosome 4B, a PH-related QTL (identified by the Xgwm495 MTA) is detected in the centromeric region of chromosome 4BS, which harbours the widely used dwarfing gene *Rht-B1.* Moreover, in the same region, three DArTs were found to be putatively under selection among the different *T. turgidum* subspecies. The MTAs detected for PH on chromosome 2BS might be comparable to the locus described by Gervais et al. [68], which might correspond to a locus that is homeoallelic to *Rht8*, which is located in chromosome 2DS.

For HD, the association mapping analysis identified MTAs in the chromosome regions of the homeologous group 2 that harbour the *ppd1* loci, which are associated to the photoperiod response, an important component of the flowering time [33]. Considering the vernalisation response, we observed MTAs on chromosome arms 5BL and 7AS in the proximity of *Vrn1* and *Vrn3*, respectively. In contrast, we did not observe any QTLs on chromosome 5A near the frost tolerance loci. All of these data can be explained by the presence in our collection of spring and intermediate cultivars, although in the absence of true winter genotypes that with few exceptions are associated to modern breeding, all of these belong to hexaploid wheat [61].

Similarly for the PC; in the same genomic region where Uauy et al. [54] mapped the PC *Gpc-B1* locus, we found one MTA on chromosome arm 6BS. Indeed, our MTA and the *Gpc-B1* locus have been mapped in the same genomic interval defined by two markers (Nor2 and Xgwm193) shared by both studies. Interestingly, this MTA is very close (1.6 cM) to a marker that is putatively under selection (wPt-7489).

The exploitation of the signature of selection on the structure of the molecular diversity [34], [69] is an alternative method to validate loci/genes previously mapped and to identify genomic regions that are involved in genetic control of important traits, even without any prior information [35], [70]–[71]. Here we have complemented the association results with the analysis of selection signatures that was performed by Laidò et al. [43], with a comparison of the various subspecies of durum wheat. Indeed, as seen in *Drosophila* by Schwarzenbacher et al. [72], the combination of genome-wide association studies with selection signature scans can increase the resolution for QTL detection. Thus, among the 45 novel QTLs, we considered only those that were identified by a locus that was putatively under selection. However, for practical breeding purposes, an analysis of the GXE interaction is needed before the implementation of an appropriate molecular breeding strategy. Considering all of the traits together, 15 novel QTLs detected in the present study are identified from MTAs putatively under selection. Most of these QTLs are specific traits, with three exceptions: one on chromosome 3A, with a QTL for HD, PC and TKW; and two on chromosome 3B, one for HD and PC, and a second for TKW and PC. The remaining 12 trait-specific QTLs were: five for PC, three for PH and TKW, and one for HD.

Considering PH, three new QTLs were identified on chromosomes 1B, 3A and 7B. In the telomeric region of the chromosome arm 1BS, the first QTL was identified in the *durum* sub-sample (*R^2^* = 7%) by the wPt-0308 MTA putatively under selection. At ∼14 cM, we detected a novel multi-trait QTL for PH and HD; in particular, for PH, Xcfd15 MTA was identified in both the whole collection and the *durum* sub-sample. In this region, using LD mapping, only Maccaferri et al. [32] have reported a QTL for TKW and KNS, which was identified by a SSR marker. The second PH-related QTL was mapped in the centromeric region of chromosome arm 3AL, where many multi-trait and single-trait MTAs were identified in the present study. However, in this region, this is the first time that a QTL for PH has been detected. On the other hand, QTLs for HD were detected by previous studies, and we cannot exclude that these data in our study are due to an indirect effect of this locus on PH. On chromosome arm 7BL in the telomeric region (258 cM), we identified one PH-related QTL that is represented by two tightly linked (0.1 cM) MTAs: wPt-6276b detected in the whole collection, and wPt-9746 detected in the *durum* sub-sample. This latter marker co-segregates with two other DArTs, all of which are identified as putatively under selection.

A new HD-related QTL in the telomeric region of chromosome 1AS was identified only in the whole collection dataset, through two MTAs detected in the LD mapping analysis that were also putatively under selection. In particular, the first of these MTAs (wPt-4735) was located on the consensus map (1AS; 4.2 cM), whereas the second one (wPt-3560) was mapped in the same position using the LD pattern (*r^2^* = 0.95).

In the present study, considering the PC trait, two novel QTLs were detected on chromosome 1B and on the group 3 chromosomes, and one on chromosome arm 7A. For the two QTLs on chromosome 1B, the first was detected in the telomeric region on chromosome arm 1BS by three MTAs, all of which were identified in the *durum* sub-sample. Among these, the wPt-5899 marker was putatively under selection, and using the LD pattern, the wPt-1876 MTA for PC, which was unmapped and putatively under selection, was located in the same position. The second QTL, in the telomeric region on chromosome arm 1BL, was identified by two MTAs, of which the wPt-8866 marker detected in the whole collection was putatively under selection. This marker is in high LD with the other two unmapped MTAs related to PC (wPt-9204 and wPt-1507), which were putatively under selection. In the telomeric regions of chromosome arms 3AL and 3BL, two PC-related QTLs were identified for the *durum* sub-sample (*R^2^* = 5% and 4%, respectively). On chromosome arm 7AL, only two co-segregating DArT markers (wPt-1976 and wPt-3403) were detected as putatively under selection, the first of which was identified as a MTA for PC.

Three novel TKW-related QTLs were detected on chromosomes 3A, 5B and 6B. On chromosome arm 3AL, a TKW-related QTL was identified in both the whole collection and the *durum* sub-sample by two MTAs, of which the wPt-1888 marker is putatively under selection. In this centromeric region of 13 cM on chromosome arm 3AL, six DArT markers were mapped, with all of these identified as MTAs for TKW, with *R^2^* from 2% to 6%. The second QTL was mapped on linkage group 5B-3, from two MTAs, of which wPt-7605 was putatively under selection. The last QTL on chromosome arm 6BS was detected by three MTAs, of which wPt-4742 was identified in both the whole collection and the *durum* sub-sample. Between these MTAs, we detected two DArT markers (wPt-5333, wPt-3309) that are putatively under selection, so this QTL was identified by two different approaches.

## Conclusions

Our data have important implications in relation to the development of association mapping studies in durum wheat, which suggests that whole-genome scan approaches are feasible. Moreover, with careful assessment of the population structure, it will be possible to choose appropriate populations to obtain different resolutions in association studies in tetraploid wheat. Additional LD studies that focus on a few genomic segments will be needed to obtain a more complete picture of the level and structure of the LD, and to conduct LD mapping based on a candidate gene approach using specific tetraploid wheat populations (e.g., the wild form).

By the genotyping of collections of accessions and cultivars with a large number of markers, association studies provide the means to improve the genetic characterisation of the germplasm. Markers associated with genomic regions that control traits of agronomic interest improve our understanding of the genetic value of the individual genotypes within germplasm collections. Moreover, as similar studies are completed, the genetic characterisation of many different germplasm sets will provide researchers with a comprehensive global perspective of the tetraploid wheat gene pool. The current study is amongst the first to assemble and analyse a population that represents all of the tetraploid wheat sub-species, among which 108 durum wheat accessions (96 mainly as elite cultivars) are representative of the Italian durum wheat breeding programmes over the last 100 years. The information that we have generated is also valuable for the choice of parental lines to use in crosses (in the form of bi-parental or MAGIC populations) to complement genome-wide association mapping data and to analyse some of the novel associations in more detail.

## Supporting Information

Figure S1 **Overview of the LD parameter** ***r^2^*** **of the intrachromosomal pairs in the single chromosome with a large number of DArT markers.** The scatterplots show the distribution of the LD parameter *r^2^* according to the genetic distance.
(PDF)

Figure S2 **Phenotypic distribution for plant height (PH), heading date (HD), protein content (PC), and thousand kernel weight (TKW) in the whole collection.**
(PDF)

Figure S3 **Phenotypic distribution for plant height (PH), heading date (HD), protein content (PC), and thousand kernel weight (TKW) in the** ***durum*** **sub-sample.**
(PDF)

Table S1 **Overview of the LD in the intrachromosomal pairs for the whole collection, the** ***durum*** **sub-sample, and the two groups (Q1 and Q2).**
(DOCX)

Table S2 **List of all of the MTAs identified for the four traits (PH, HD, PC, TKW) using the GLM and MLM.**
(XLSX)
