# Supplementary material for: Linkage Disequilibrium and Genome-Wide Association Mapping in Tetraploid Wheat (Triticum turgidum L.)
Source: PLoS One. 2014 Apr 23;9(4):e95211. doi: 10.1371/journal.pone.0095211 (PMC3997356; doi:10.1371/journal.pone.0095211)

Entire Collection

230 lines, 592 mapped DArTs (10% cutoff)

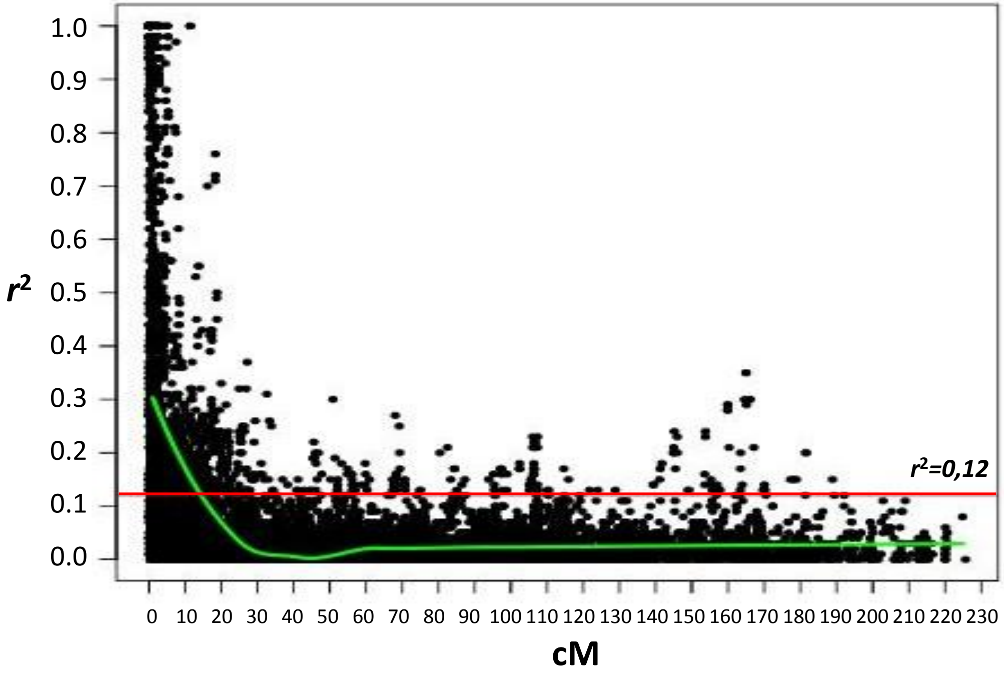

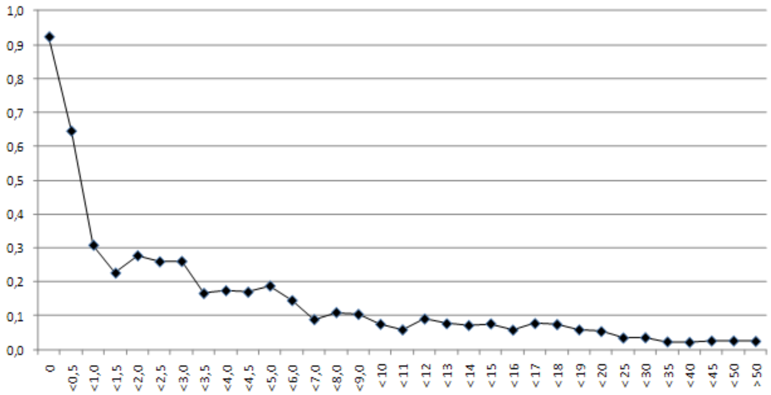

LD Intra-chromosomal

r^2=0,083 D'=0,330

LD Inter-chromosomal r^2=0,023 D'=0,217

Tot. LD r^2=0,027 D'=0,225

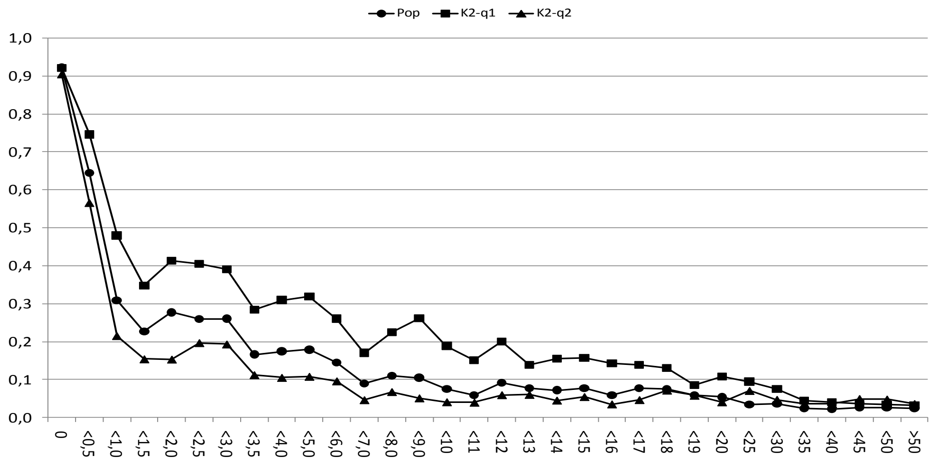

In the entire collection under investigation 5,066 (41.2%) of the 12,301 intrachromosomal marker pairs showed a significant level of LD (P<0,01). The average of r^2 for all pairs was 0.083.

Durum Wheat Sub-Sample

128 lines, 592 mapped DArTs (10% cutoff)

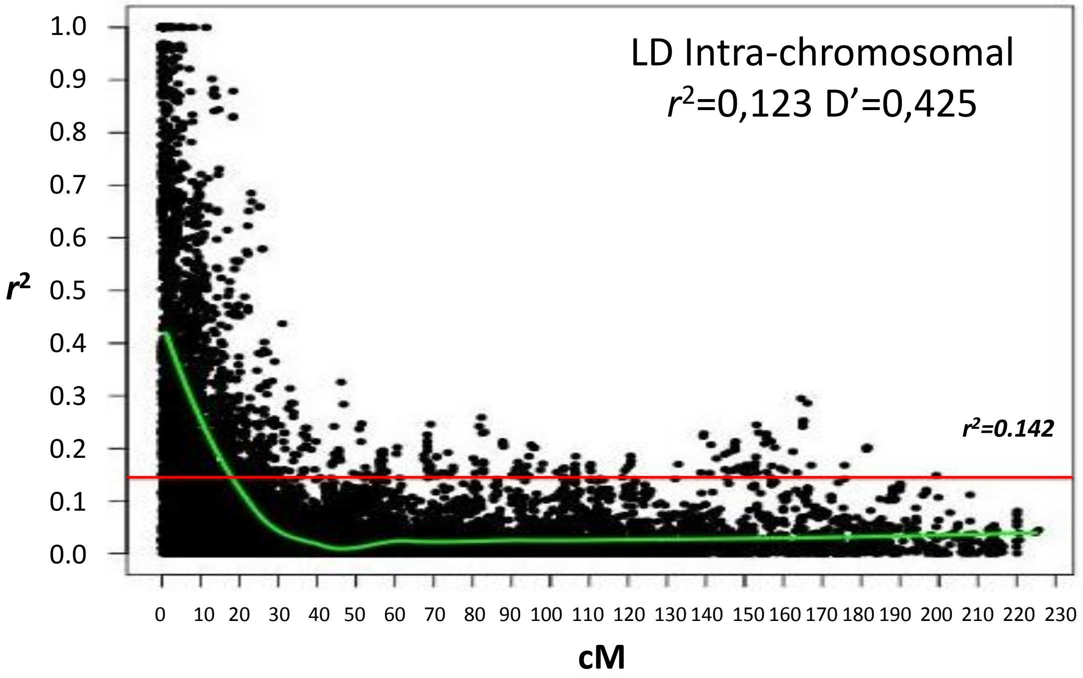

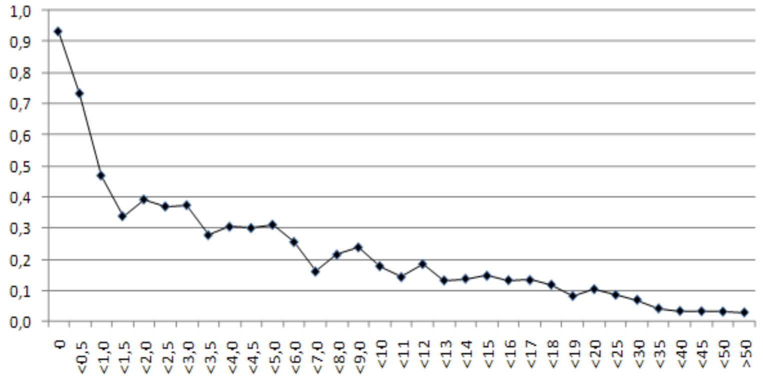

LD Inter-chromosomal r^2=0,029 D'=0,275
Tot. LD r^2=0,035 D'=0,285

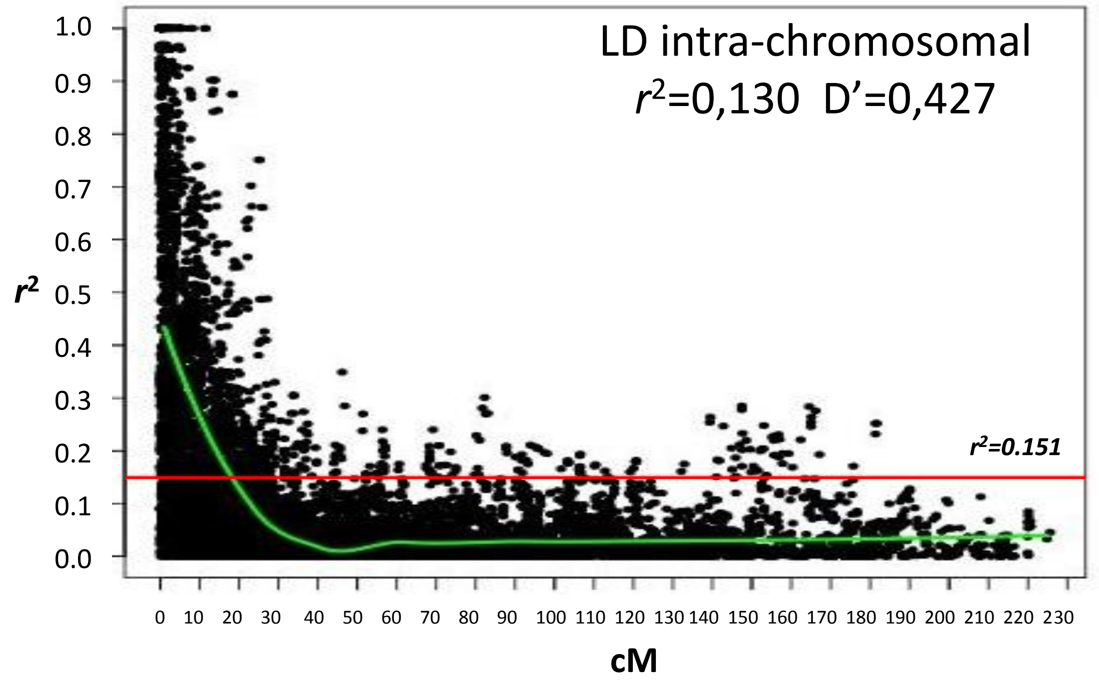

1° Sub-Group (SSR Structure)

129 lines, 577 mapped DArTs (10% cutoff)

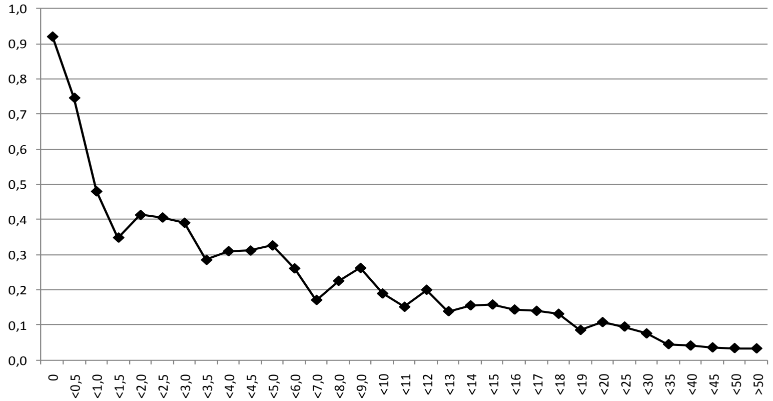

LD Inter-chromosomal LD r^2=0,031 D'=0,271

Tot. LD r^2=0,038 D'=0,282

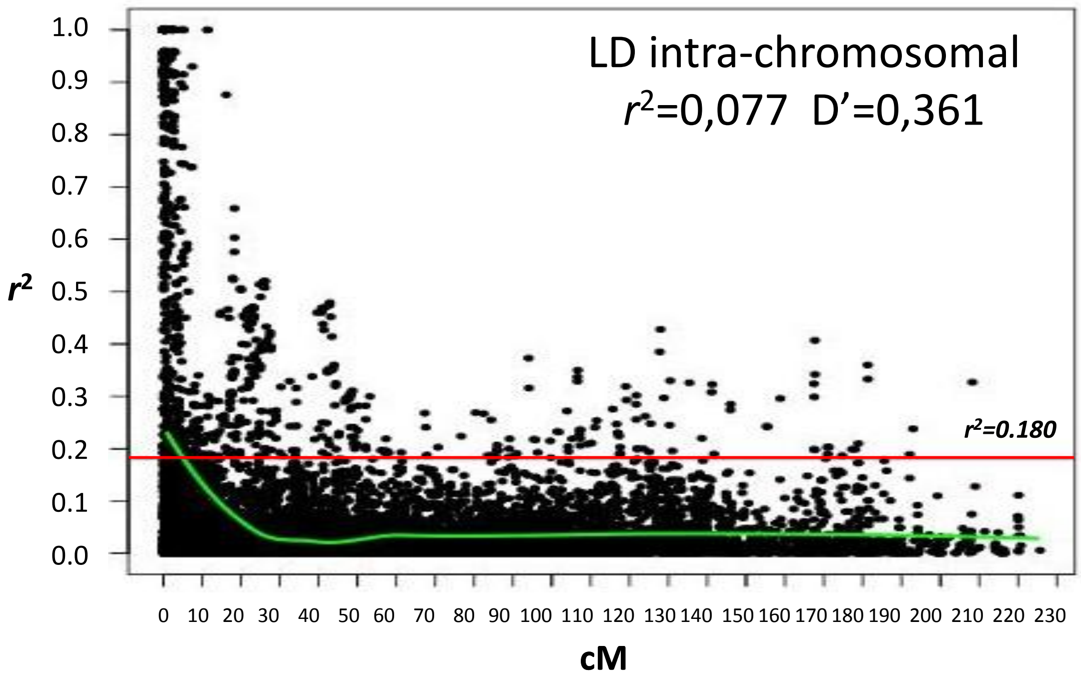

2° Sub-Group (SSR Structure)

101 lines, 565 mapped DArTs (10% cutoff)

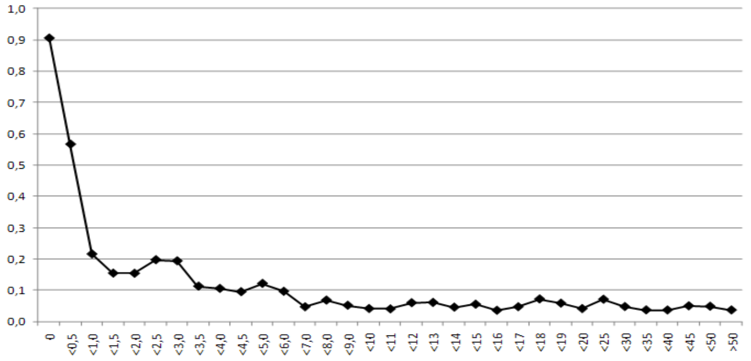

LD Inter-chromosomal r^2=0,036 D'=0,316

Tot. LD r^2=0,039 D'=0,319

Cromosoma 1B

LD Intra-chromosomal

r^2=0,06 D'=0,290

N° pairs 1378 (53 DArT markers)

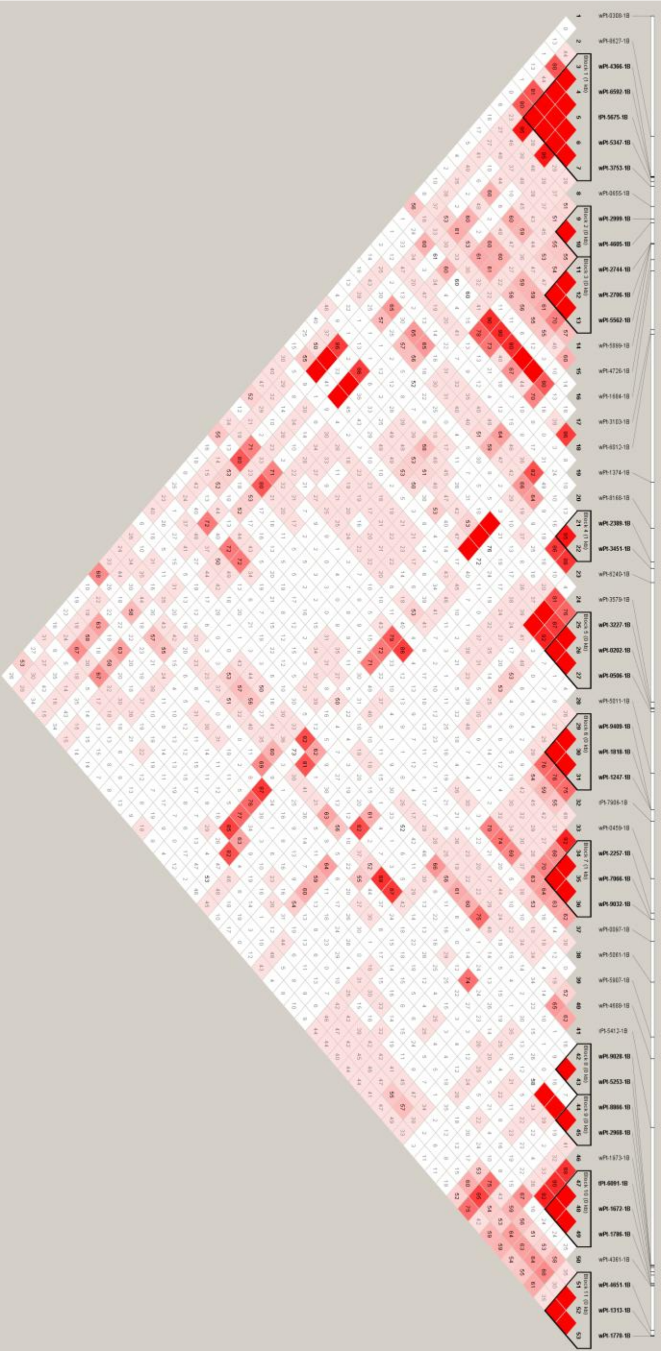

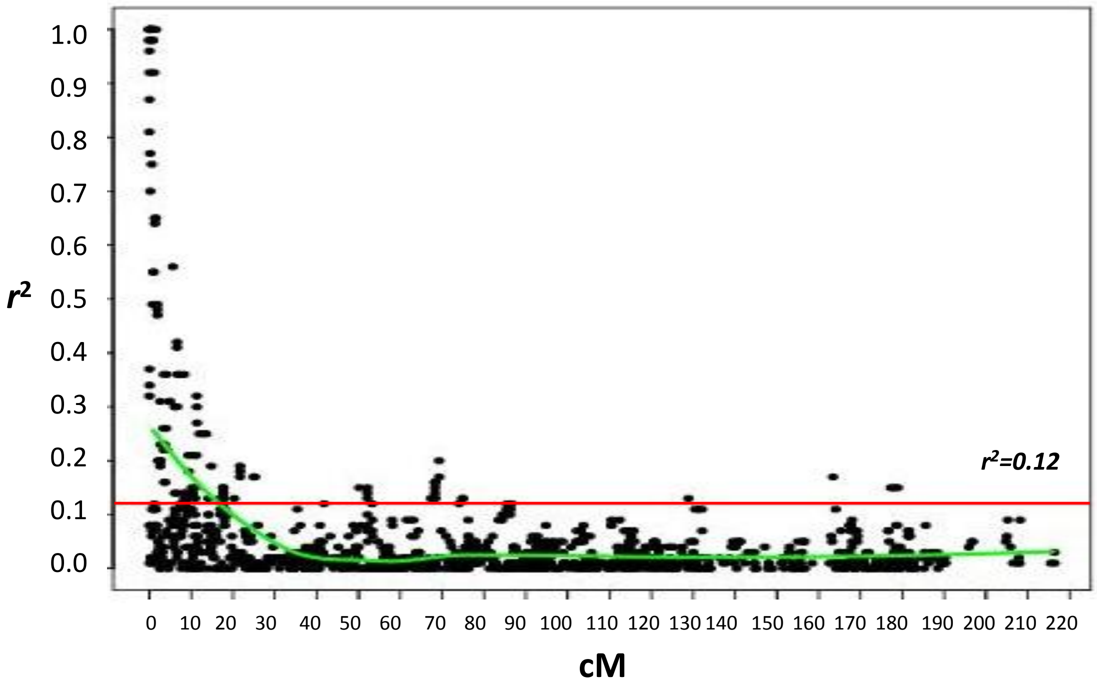

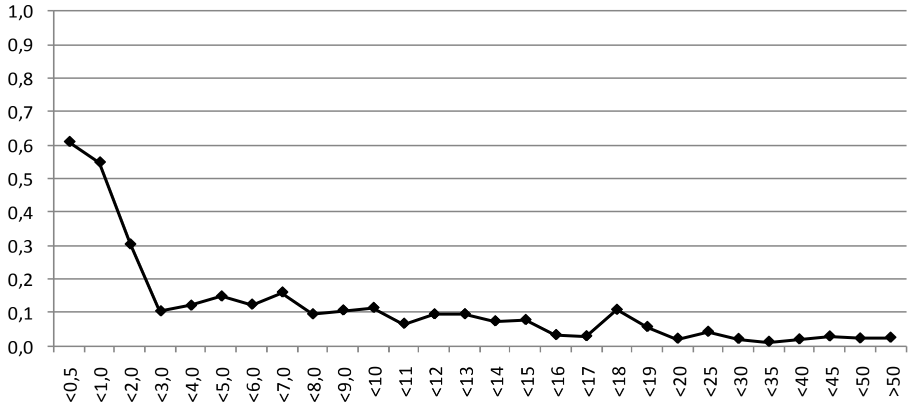

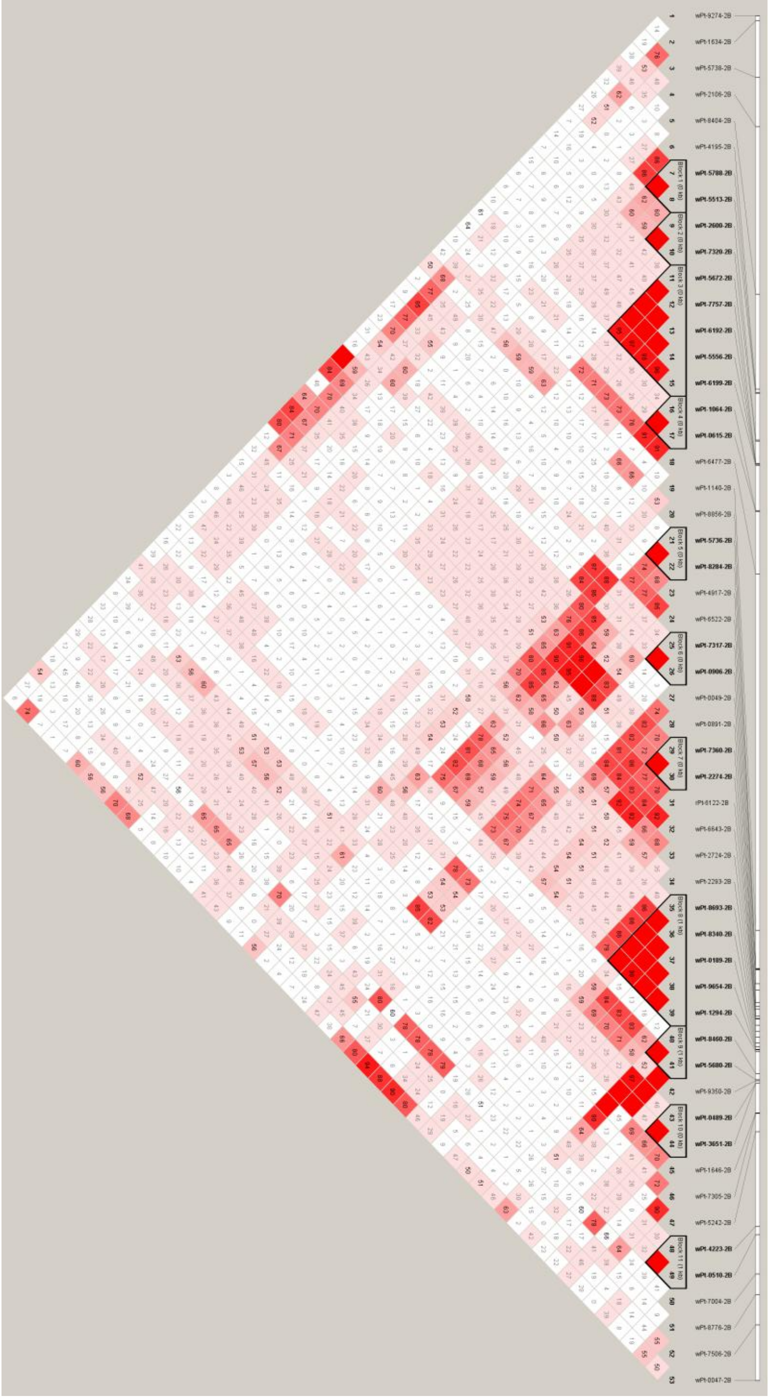

Cromosoma 2B

Mean intra-chromosomal LD

r^2=0,08 and D'=0,300

N° pairs 1378 (53 DArT markers)

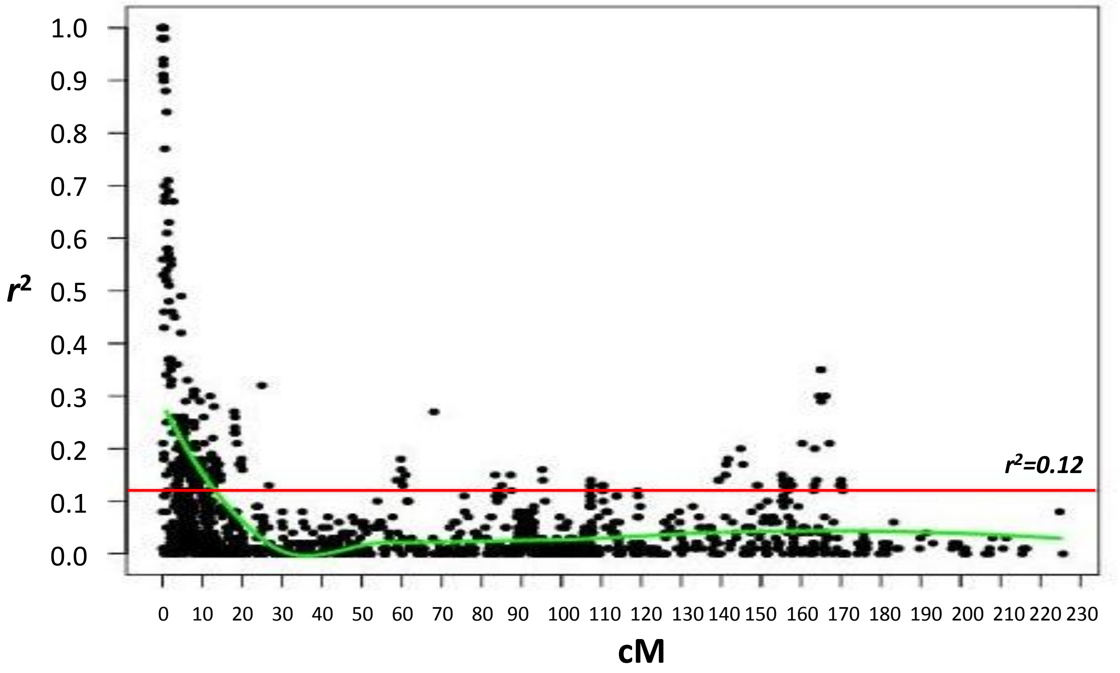

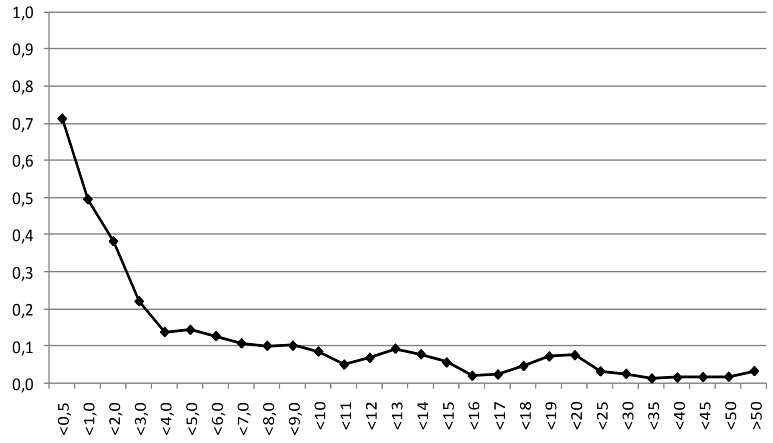

Cromosoma 3B-1

Mean intra-chromosomal LD

r^2=0,09 and D'=0,340

N° pairs 1128 (48 DArT markers)

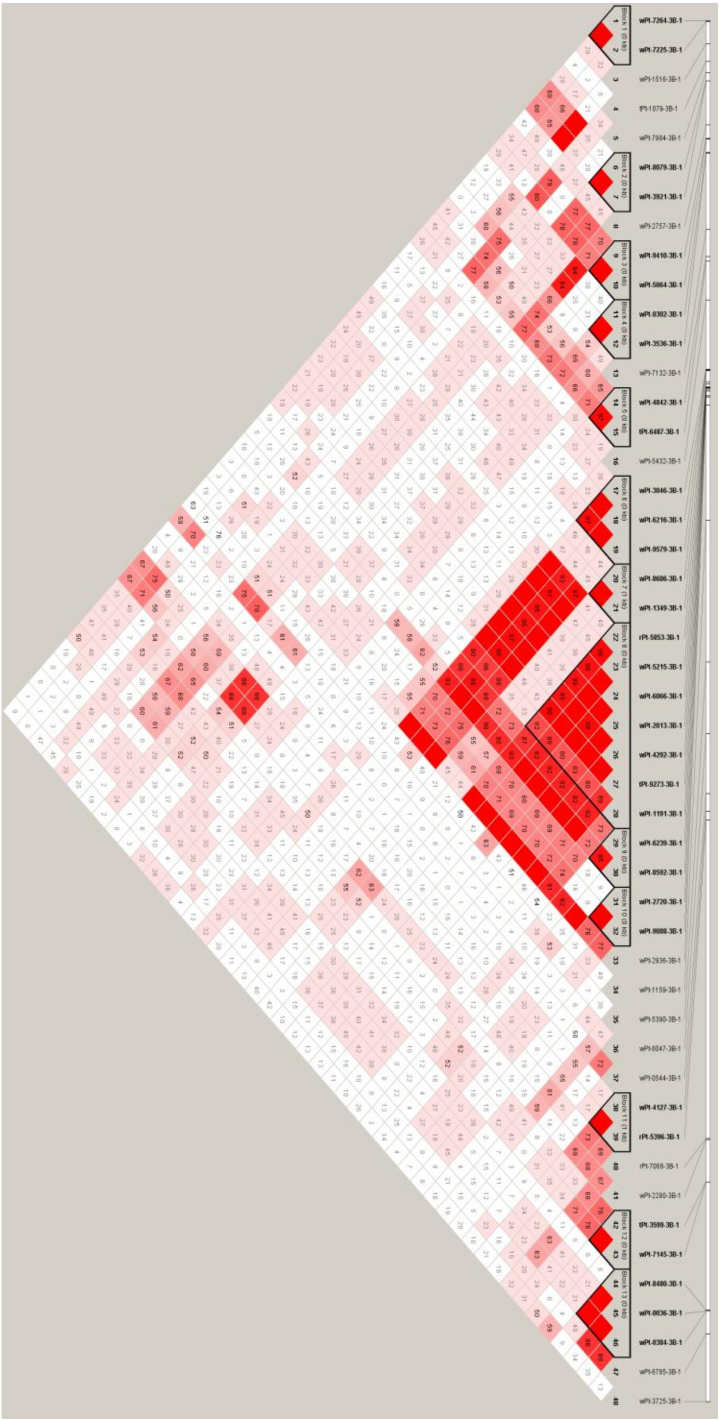

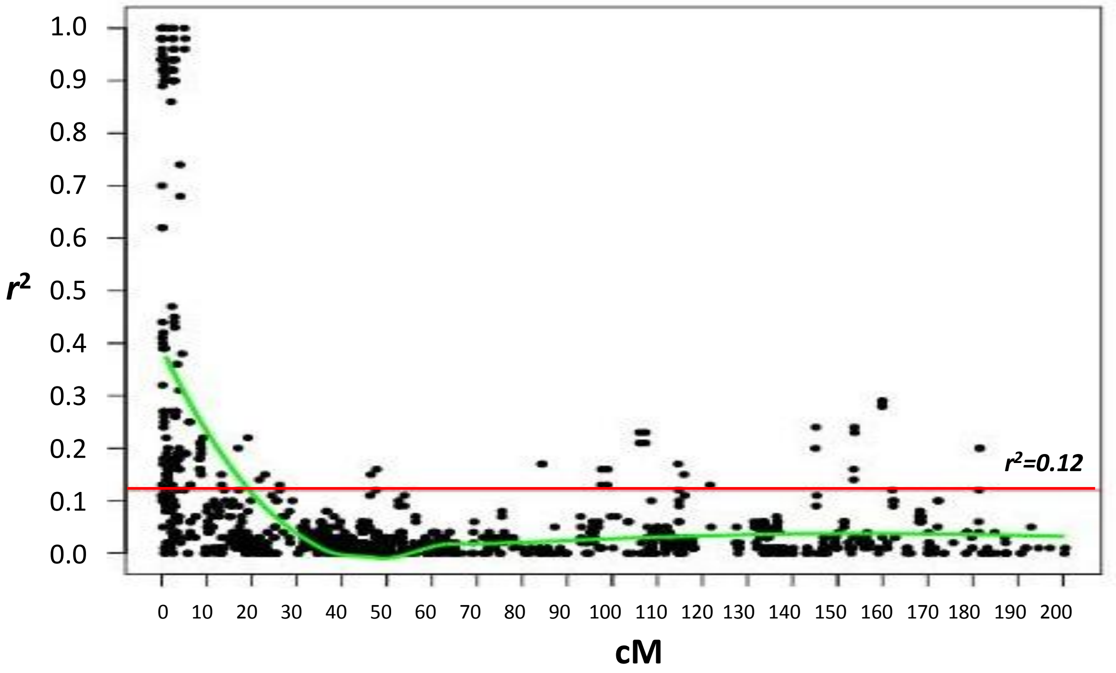

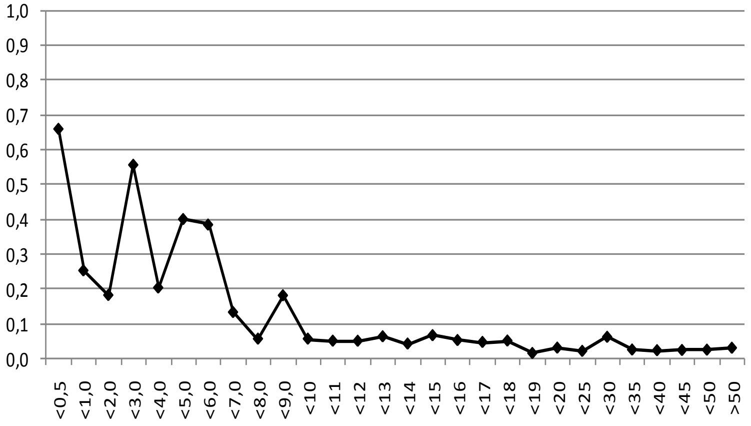

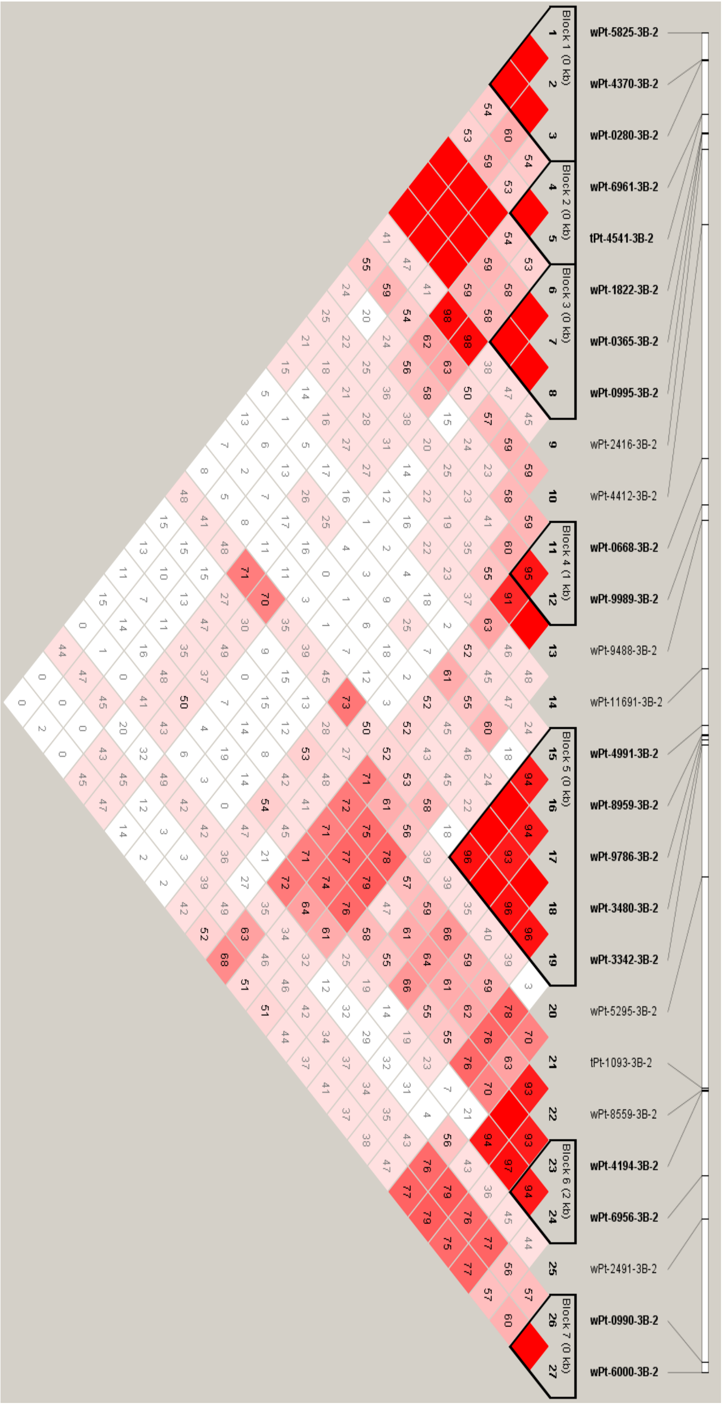

Cromosoma 3B-2

Mean intra-chromosomal LD

r^2=0,17 and D'=0,430

N° pairs 351 (27 DArT markers)

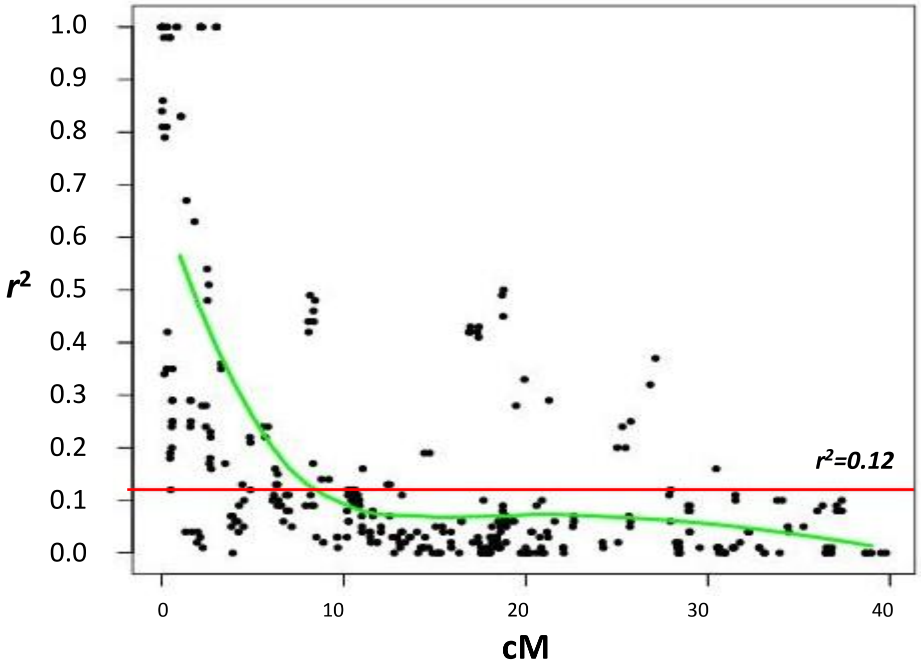

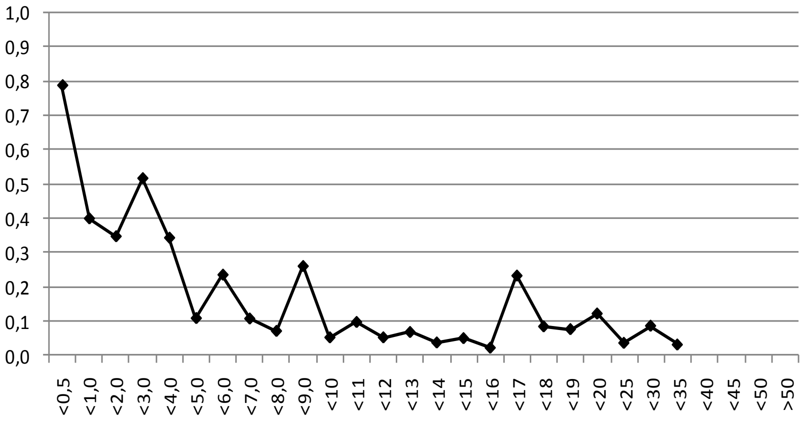

Cromosoma 4A

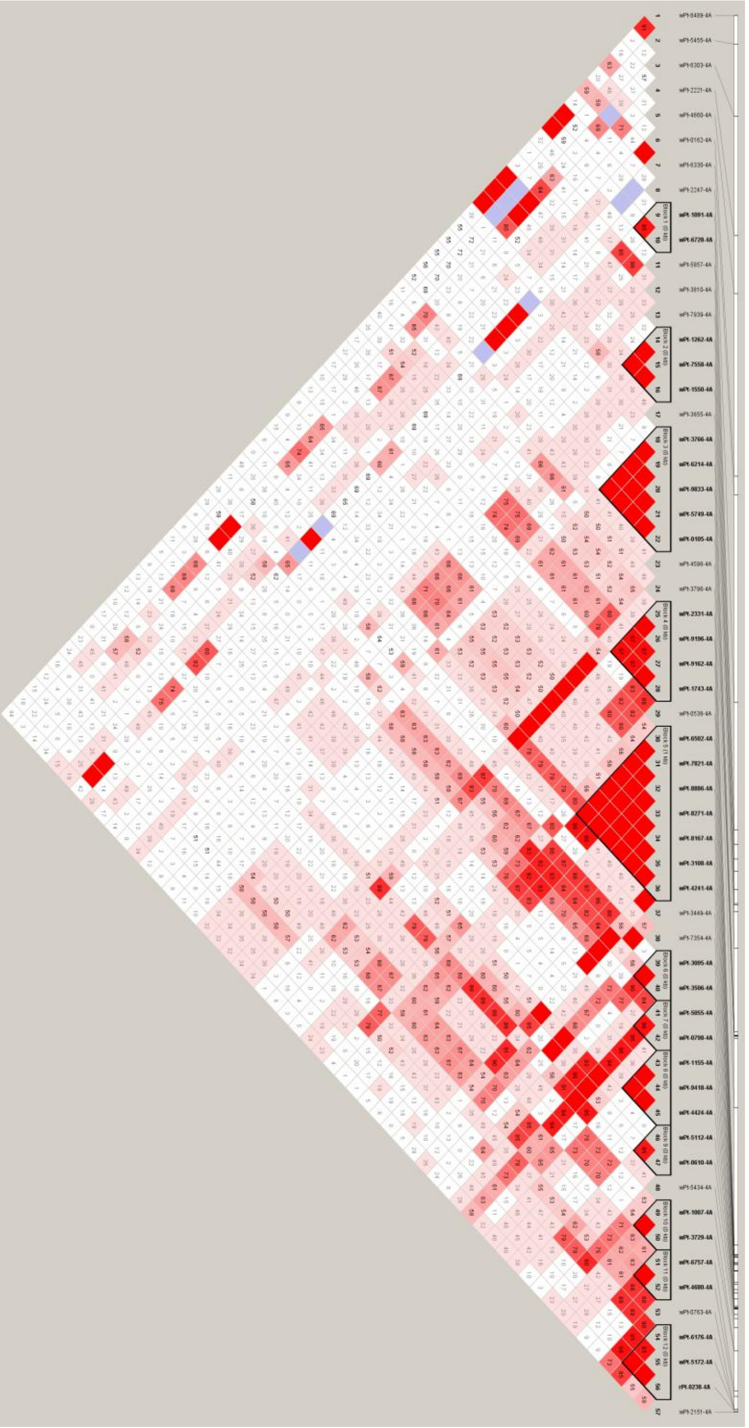

Mean intra-chromosomal LD
r^2=0,07 and D'=0,360
N° pairs 1596 (57 DArT markers)

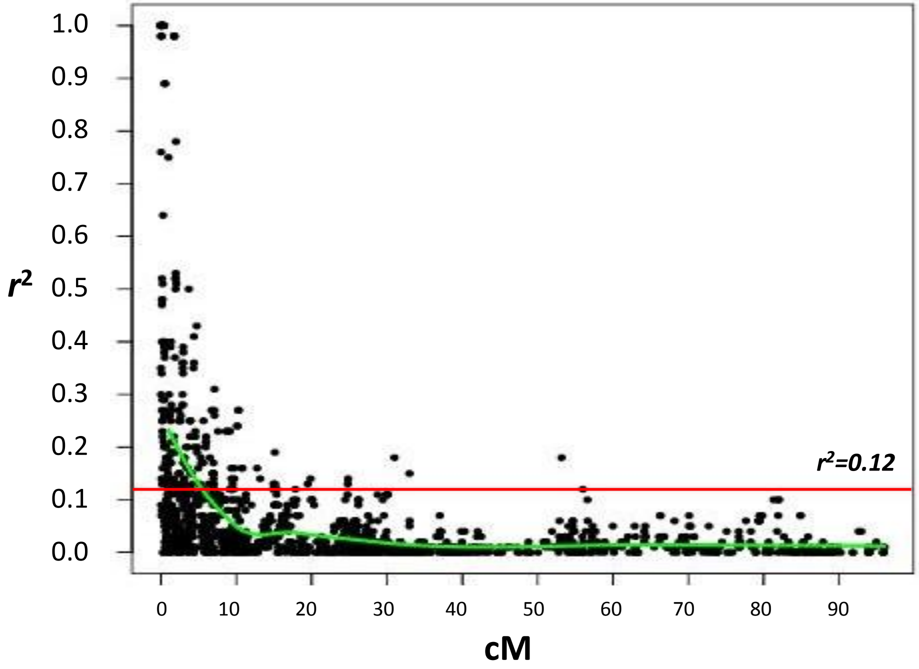

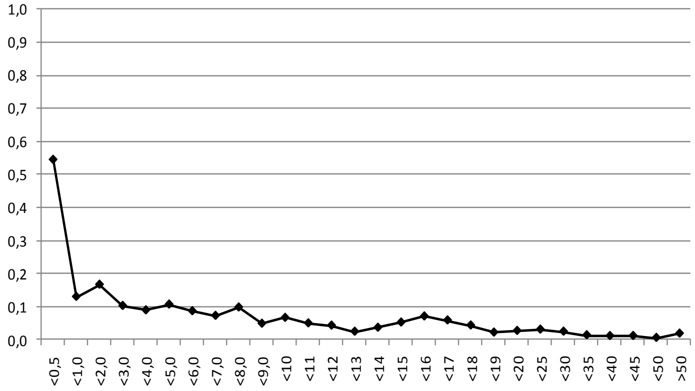

Cromosoma 6A

Mean intra-chromosomal LD

r^2=0,11 and D'=0,340

N° pairs 1486 (55 DArT markers)

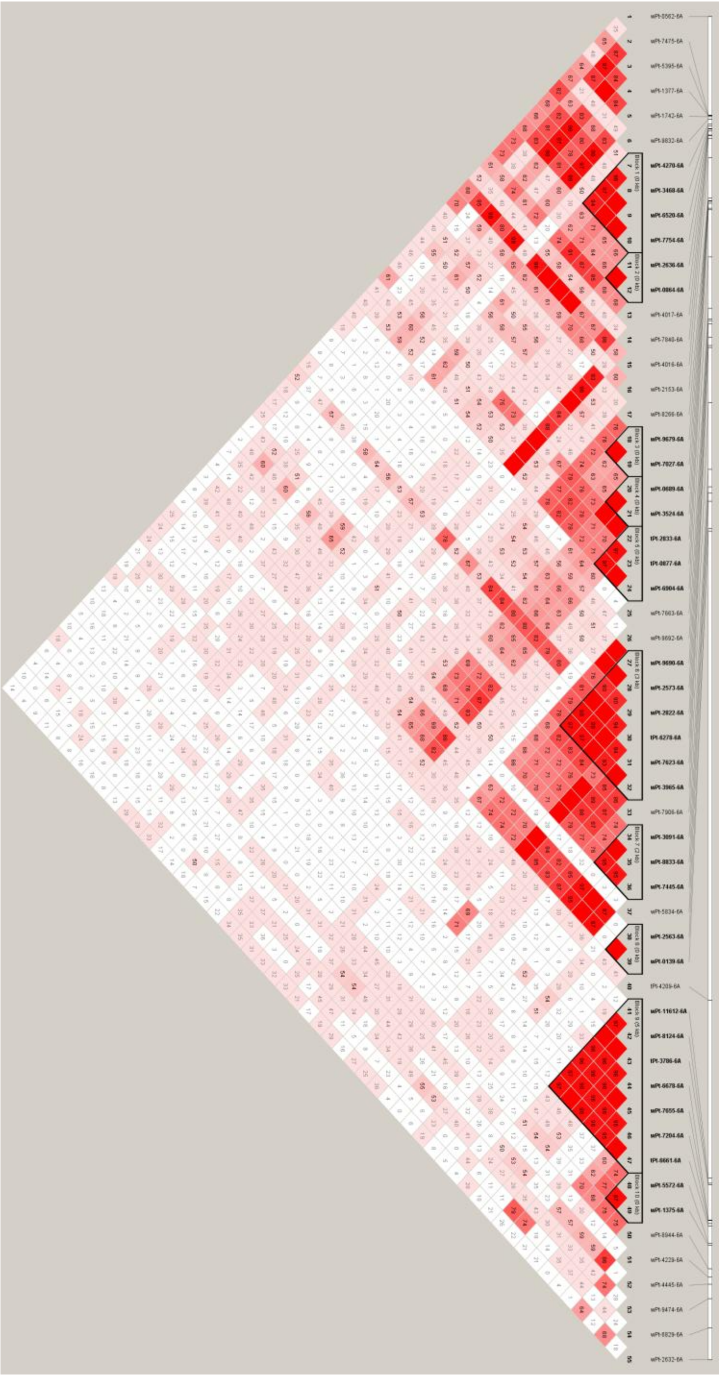

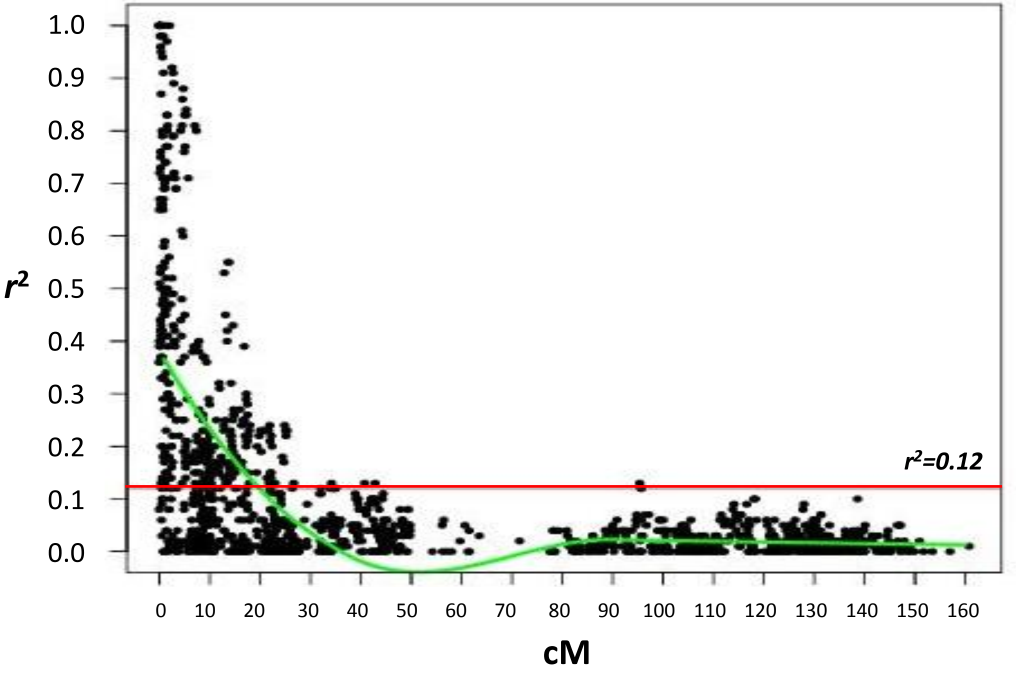

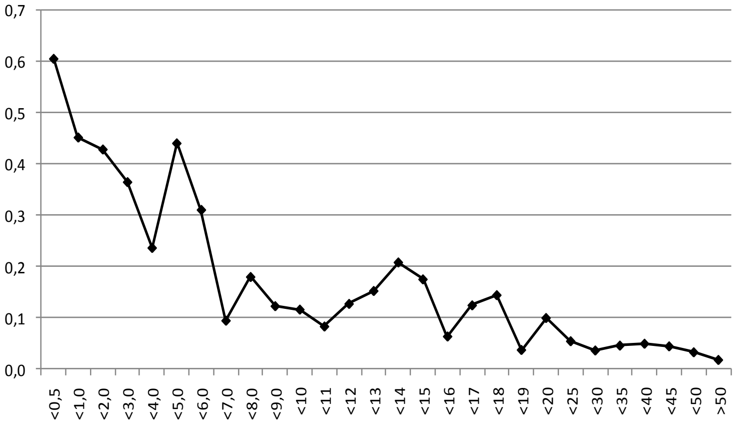

Cromosoma 6B

Mean intra-chromosomal LD
r^2=0,06 and D'=0,290
N° pairs 1769 (60 DArT markers)

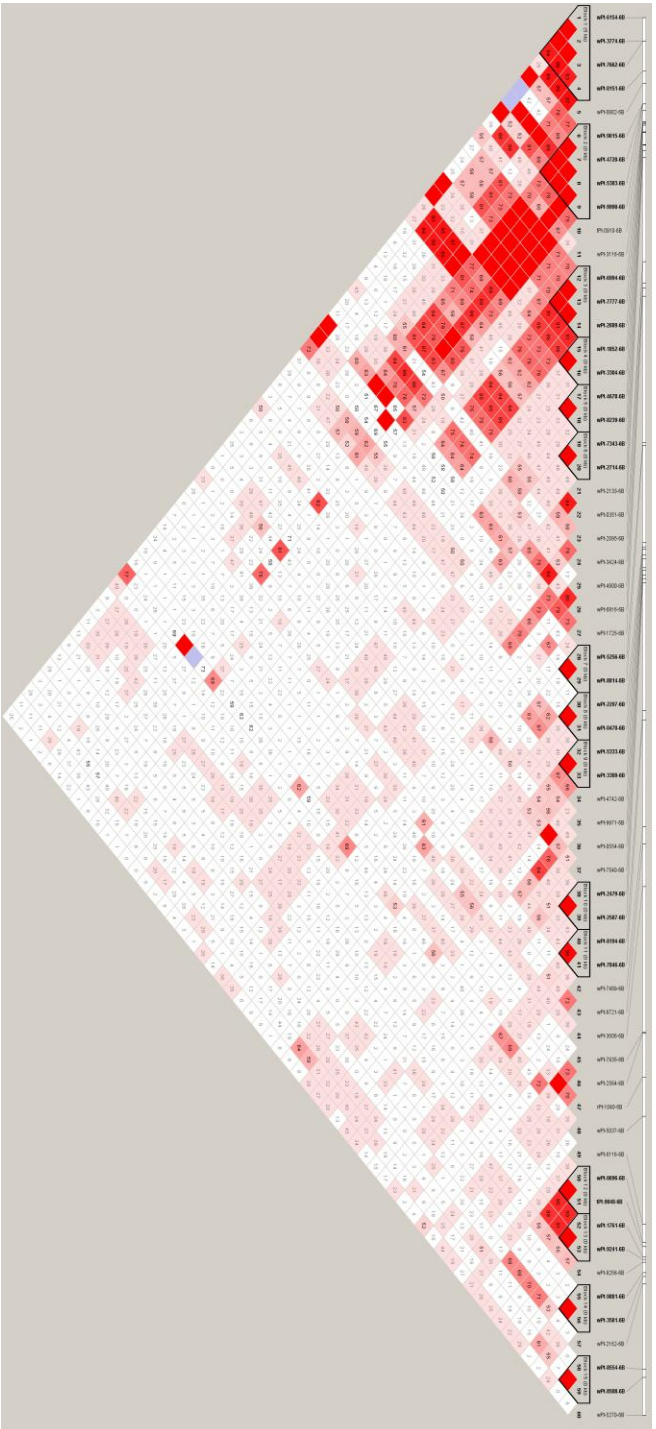

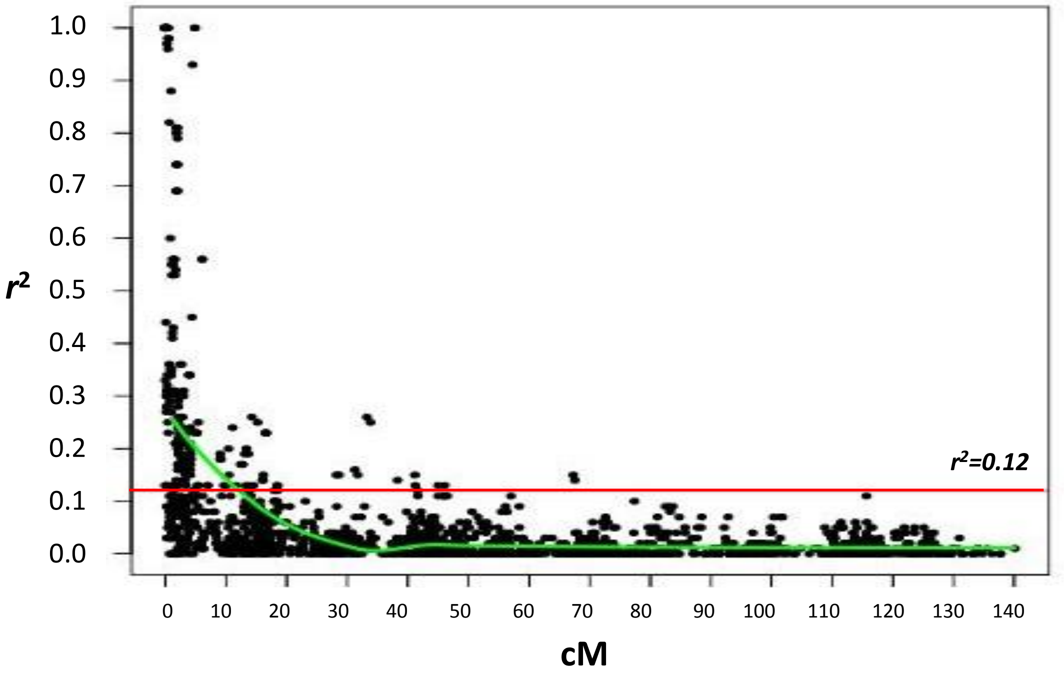

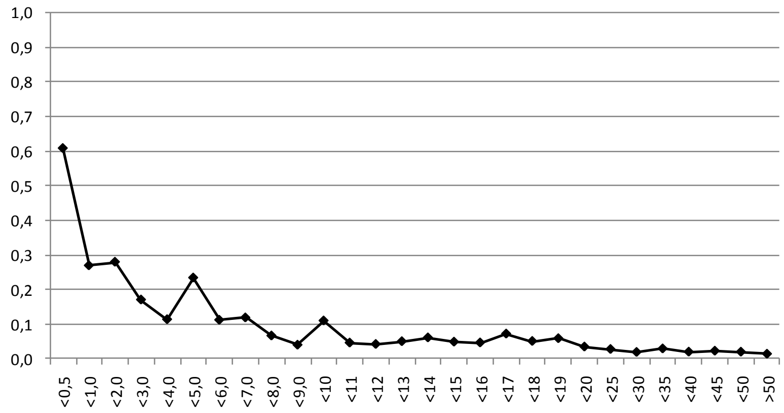

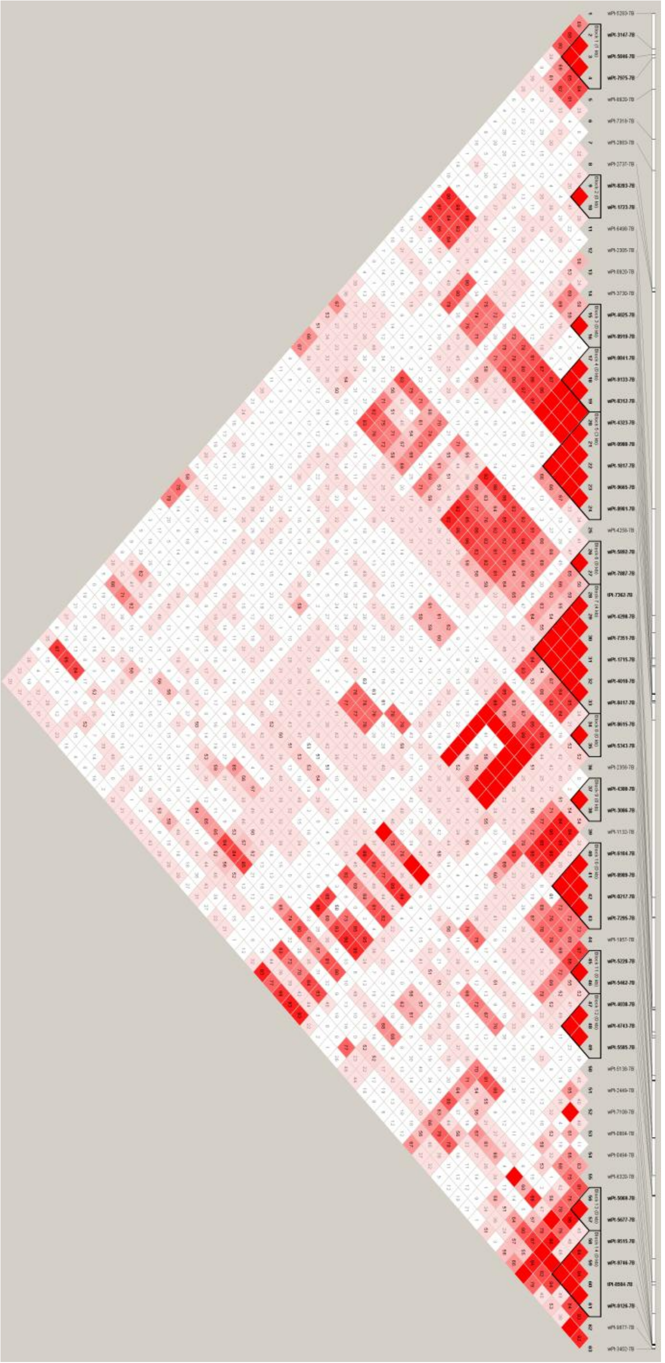

Cromosoma 7B

Mean intra-chromosomal LD

r^2=0,06 and D'=0,340

N° pairs 1953 (63 DArT markers)

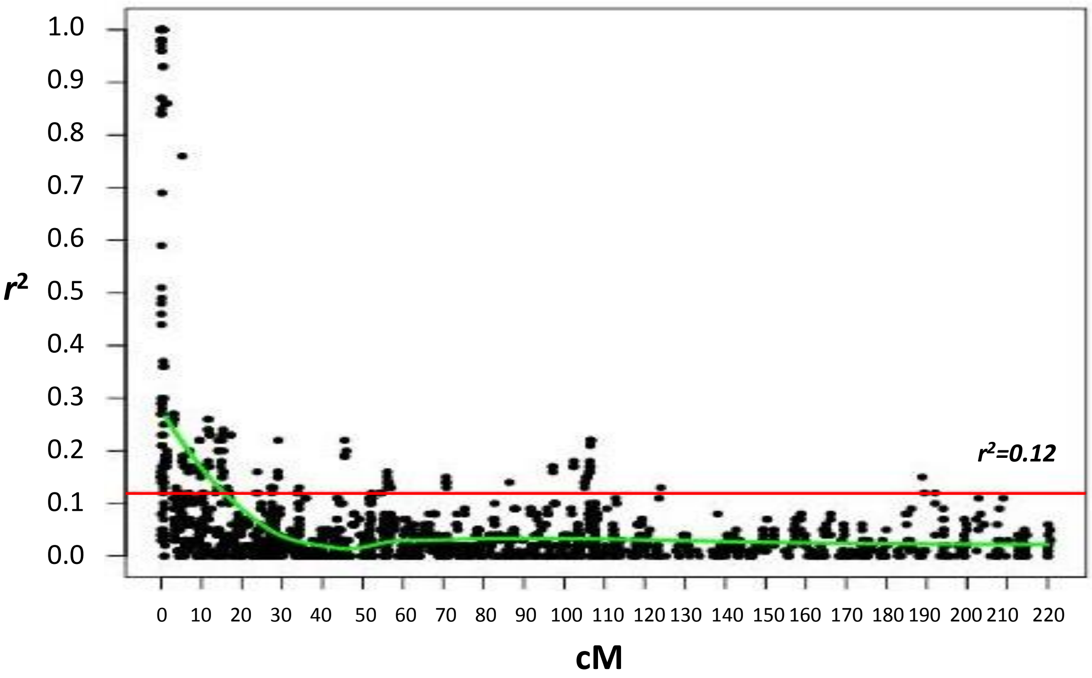

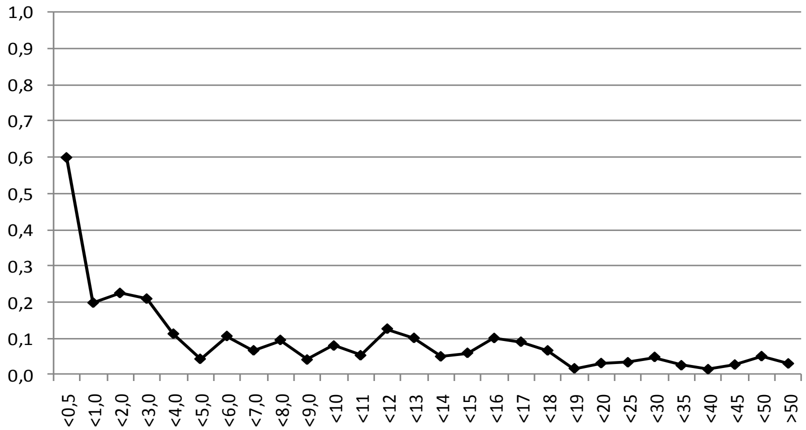

Supplement: Figure S1 — Overview of the LD parameter r2 of the intrachromosomal pairs in the single chromosome with a large number of DArT markers. The scatterplots show the distribution of the LD parameter r2 according to the genetic distance. (PDF) [file pone.0095211.s001.pdf]
