# Supplementary material for: Linkage Disequilibrium and Genome-Wide Association Mapping in Tetraploid Wheat (Triticum turgidum L.)
Source: PLoS One. 2014 Apr 23;9(4):e95211. doi: 10.1371/journal.pone.0095211 (PMC3997356; doi:10.1371/journal.pone.0095211)

Whole Collection

Summary: PH

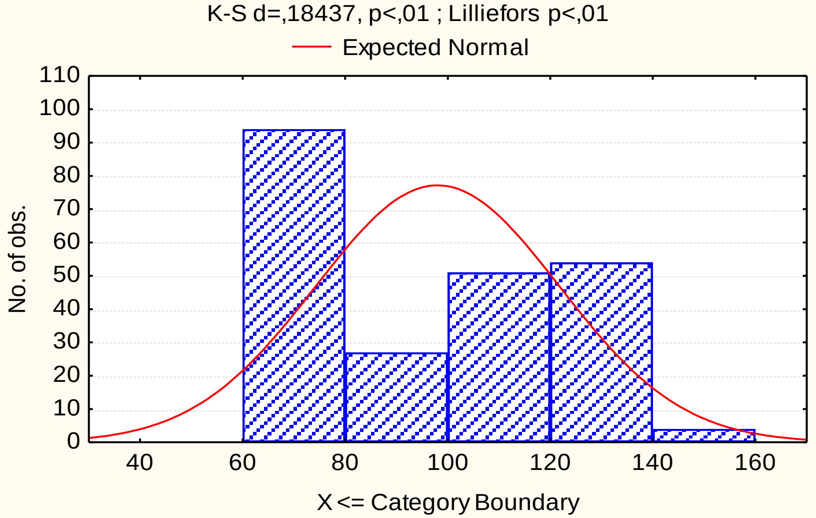

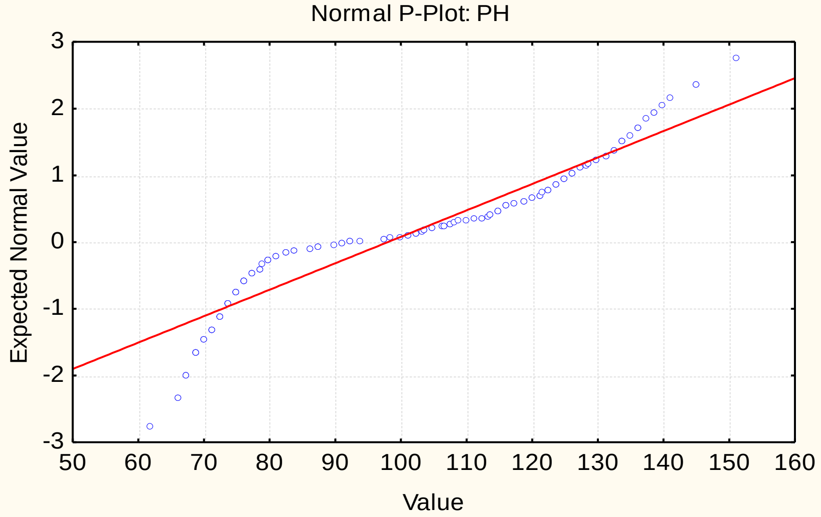

Summary Statistics:PH
Valid N=230
Mean= 98,022464
Geometric Mean= 95,198415
Minimum= 62,000000
Maximum=151,250000
Std.Dev.= 23,786454
Skewness= 0,290181
Kurtosis= -1,394506

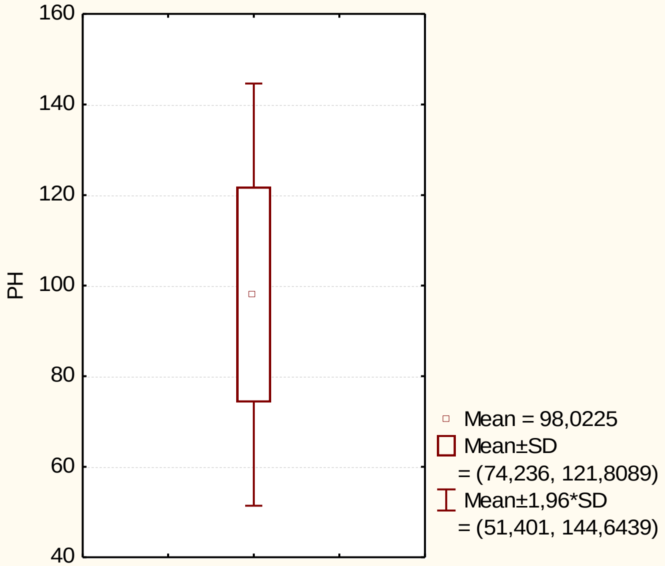

Whole Collection

Summary: HD

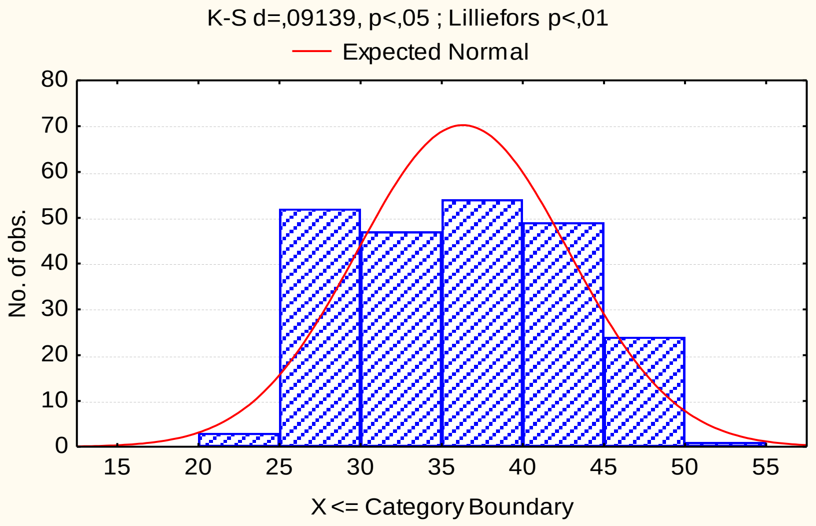

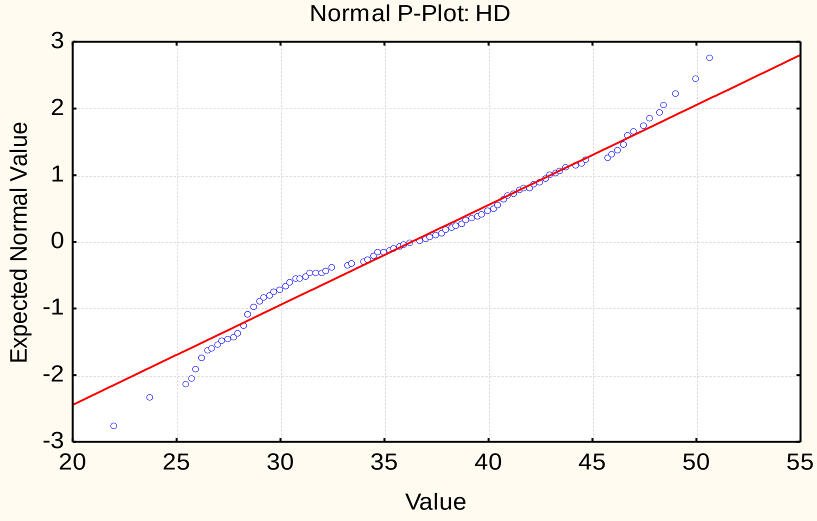

Summary Statistics:HD
Valid N=230
Mean= 36,309420
Geometric Mean= 35,715975
Minimum= 22,000000
Maximum= 50,666667
Std.Dev.= 6,531153
Skewness= 0,085635
Kurtosis= -0,966602

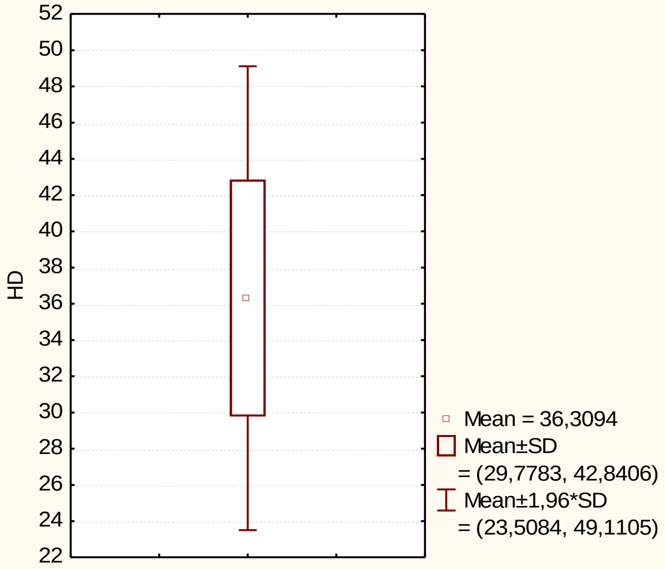

Whole Collection

Summary: PC

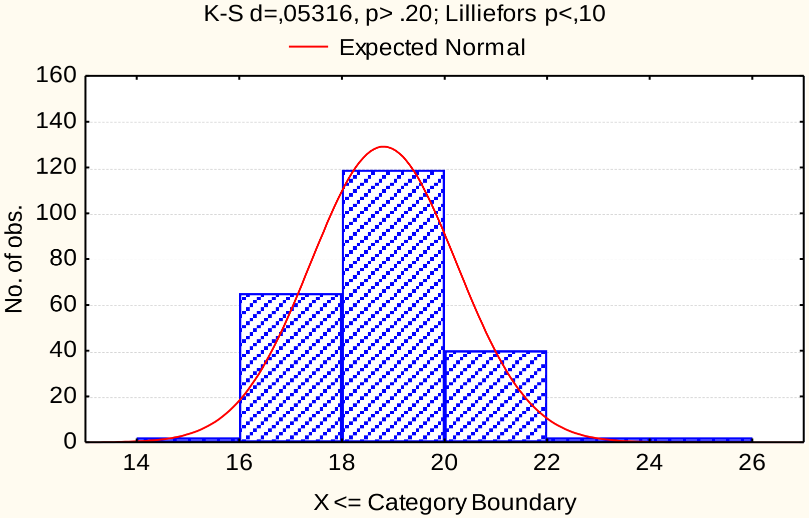

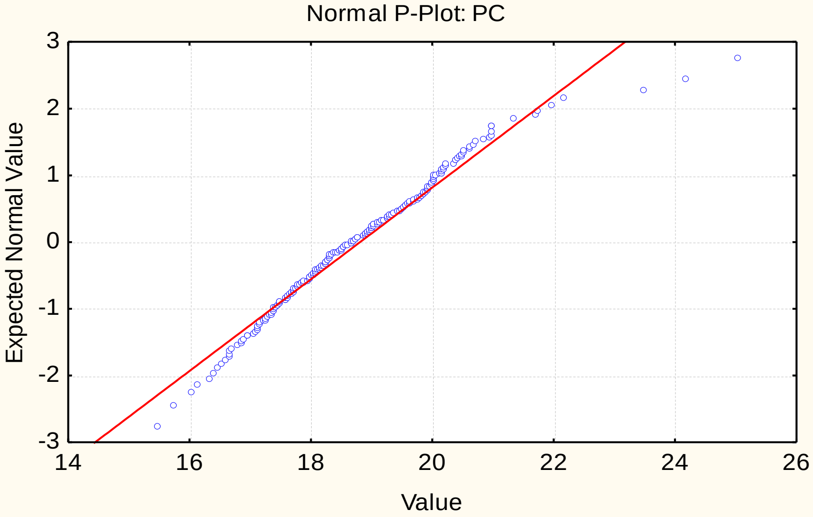

Summary Statistics:PC
Valid N=230
Mean= 18,811841
Geometric Mean= 18,759780
Minimum= 15,473333
Maximum= 25,060000
Std.Dev.= 1,420777
Skewness= 0,719361
Kurtosis= 1,799924

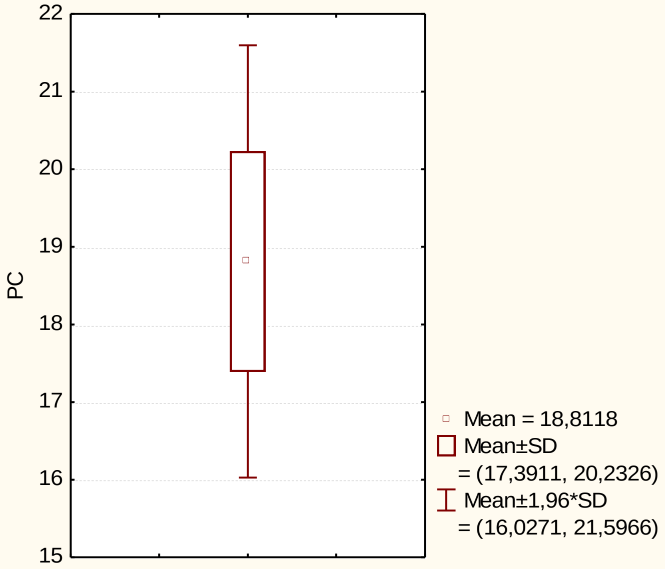

Whole Collection

Summary: TKW

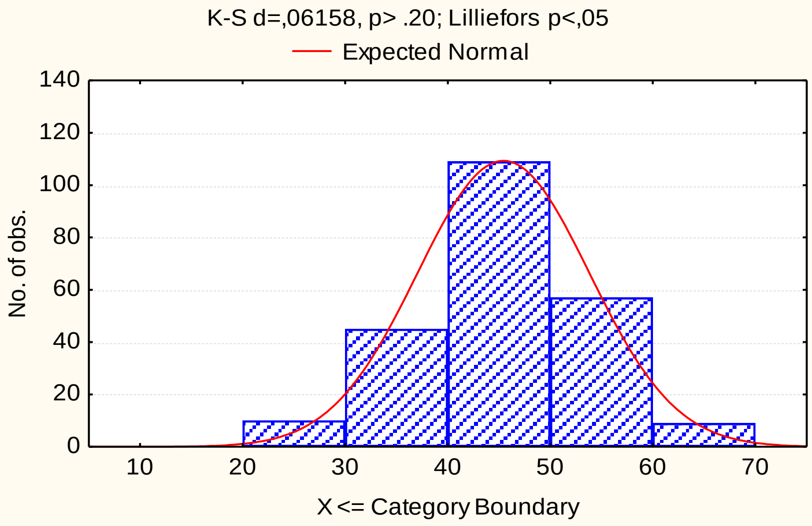

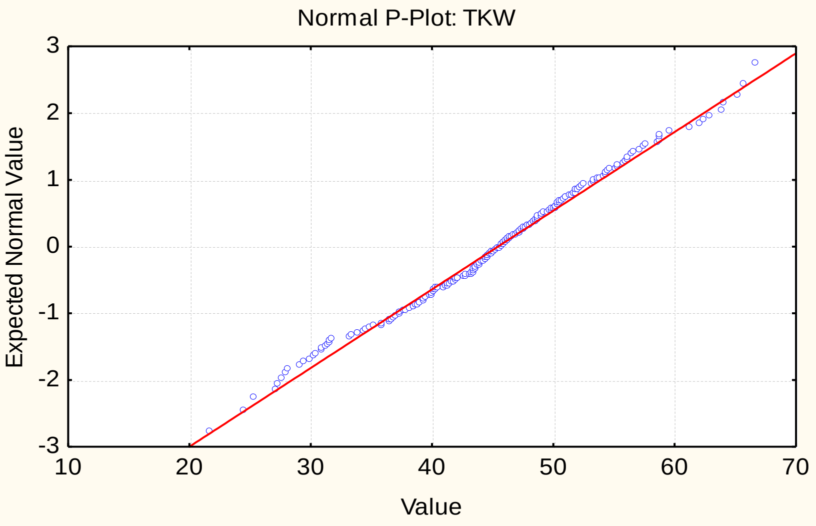

Summary Statistics:TKW
Valid N=230
Mean= 45,436377
Geometric Mean= 44,610781
Minimum= 21,750000
Maximum= 66,775000
Std.Dev.= 8,394825
Skewness= -0,143982
Kurtosis= 0,127209

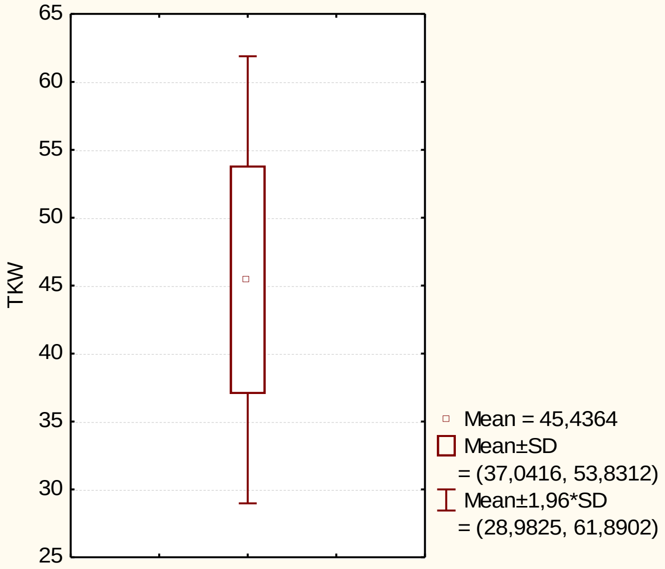

Supplement: Figure S2 — Phenotypic distribution for plant height (PH), heading date (HD), protein content (PC), and thousand kernel weight (TKW) in the whole collection. (PDF) [file pone.0095211.s002.pdf]
