# Supplementary material for: Linkage Disequilibrium and Genome-Wide Association Mapping in Tetraploid Wheat (Triticum turgidum L.)
Source: PLoS One. 2014 Apr 23;9(4):e95211. doi: 10.1371/journal.pone.0095211 (PMC3997356; doi:10.1371/journal.pone.0095211)

Durum sub-samples

Summary: PH

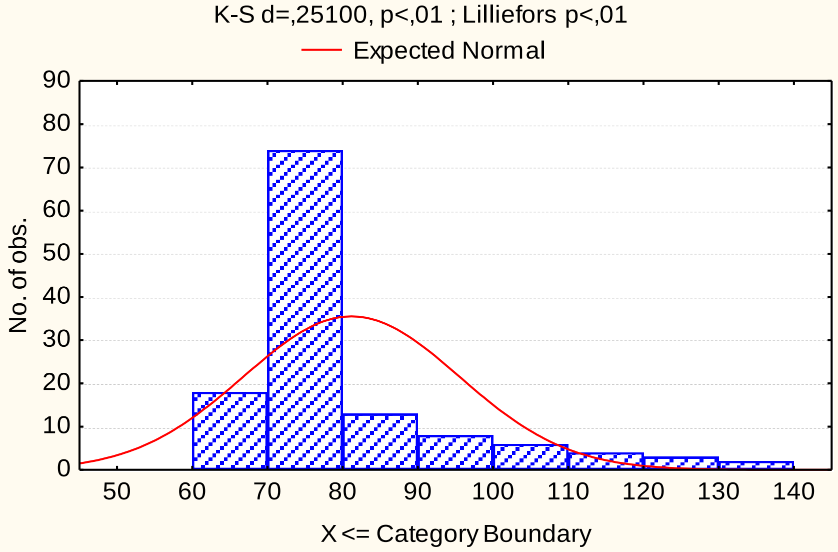

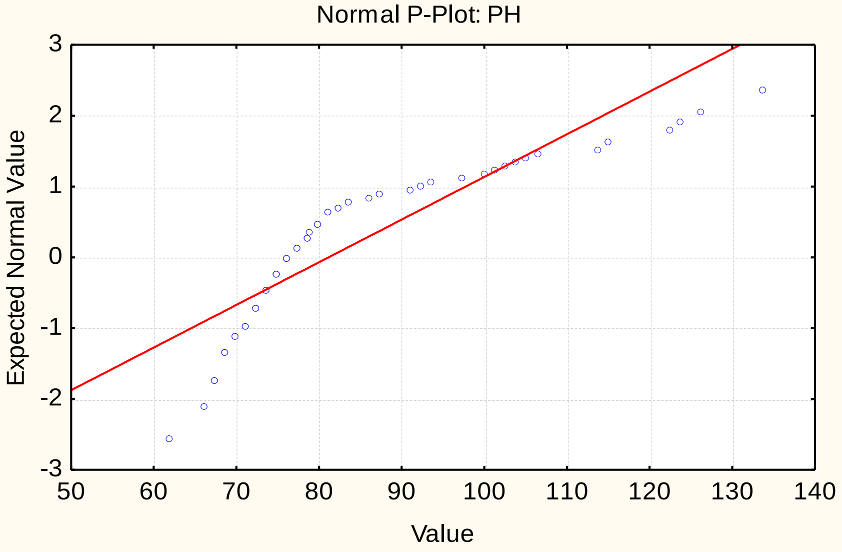

Summary Statistics:PH
Valid N=128
Mean= 81,163411
Minimum= 62,000000
Maximum=133,750000
Std.Dev.= 14,374778
Skewness= 1,904138
Kurtosis= 3,379967

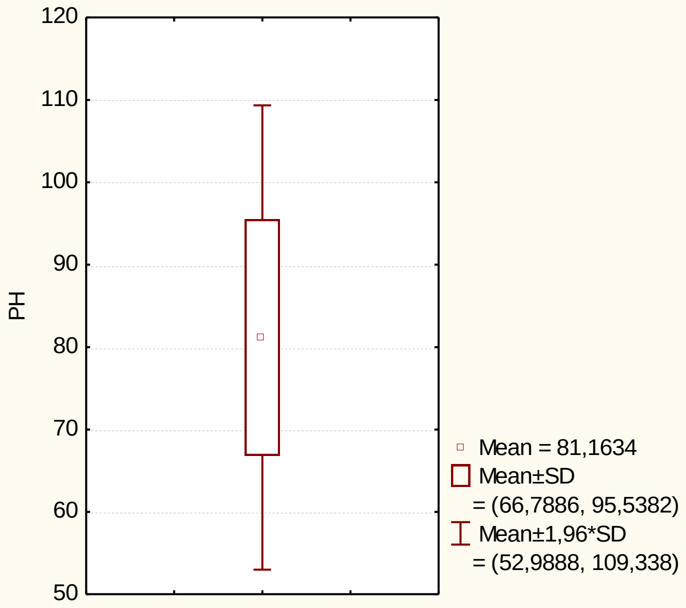

Durum sub-samples

Summary: HD

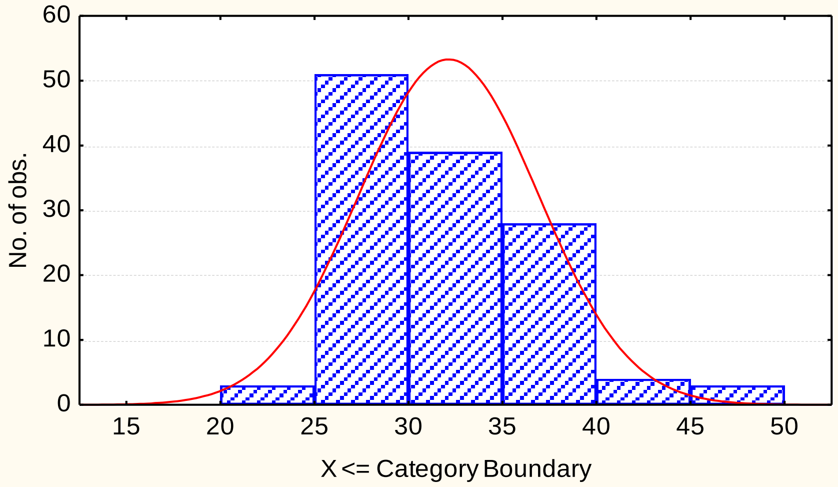

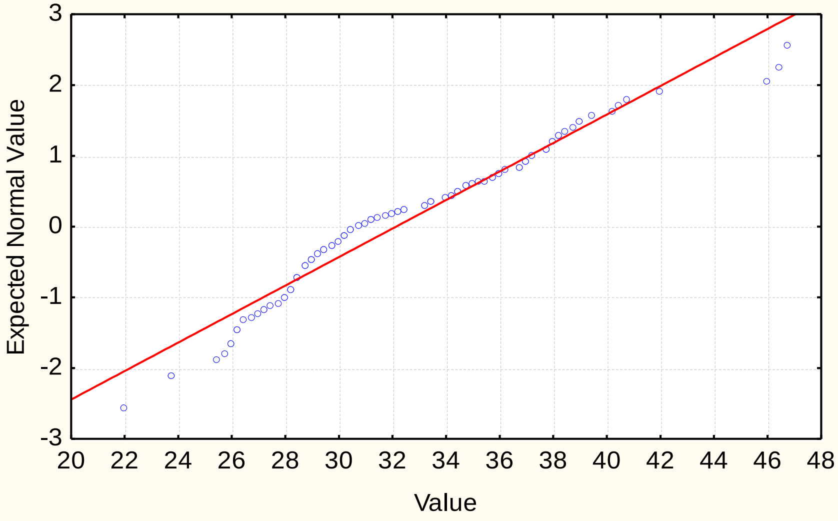

Summary Statistics:HD
Valid N=128
Mean= 32,138672
Minimum= 22,000000
Maximum= 46,750000
Std.Dev.= 4,790781
Skewness= 0,696161
Kurtosis= 0,315723

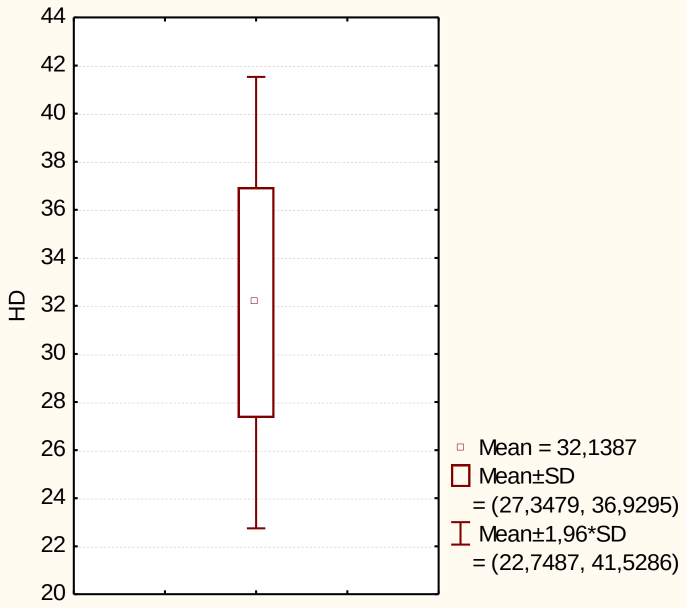

Durum sub-samples

Summary: PC

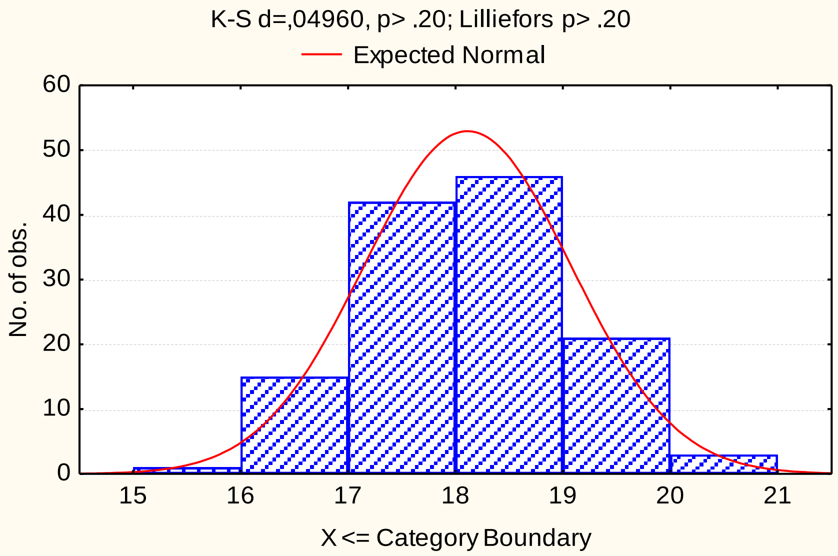

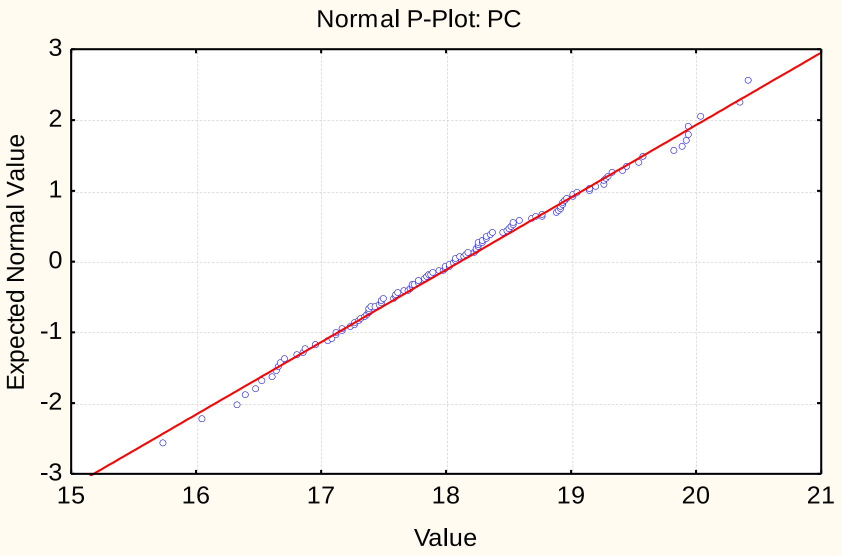

Summary Statistics:PC
Valid N=128
Mean= 18,112995
Minimum= 15,742500
Maximum= 20,420000
Std.Dev.= 0,964466
Skewness= 0,140852
Kurtosis= -0,339921

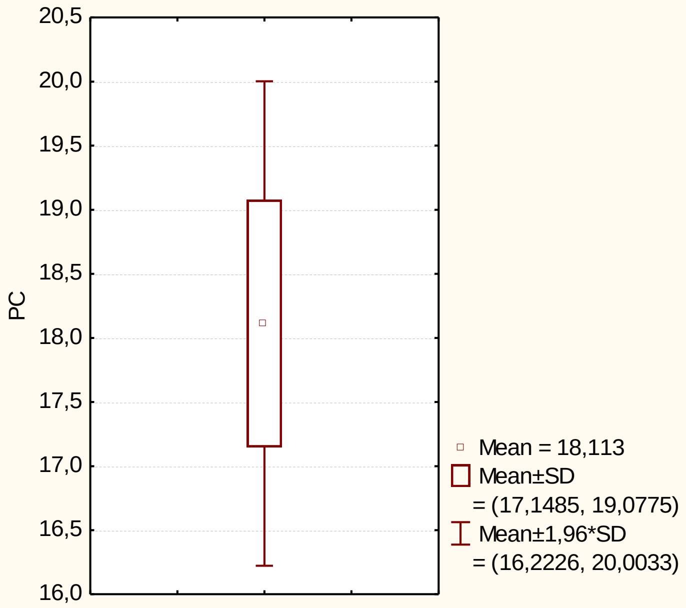

Durum sub-samples

Summary: TKW

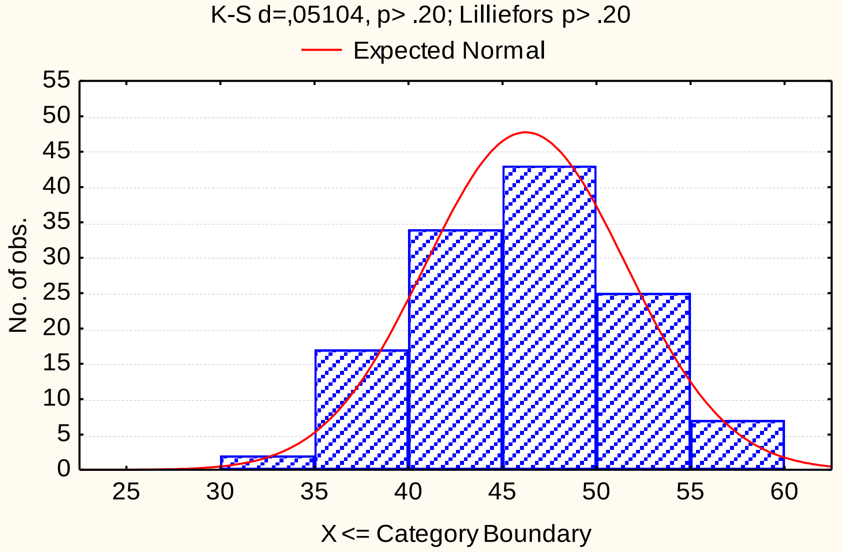

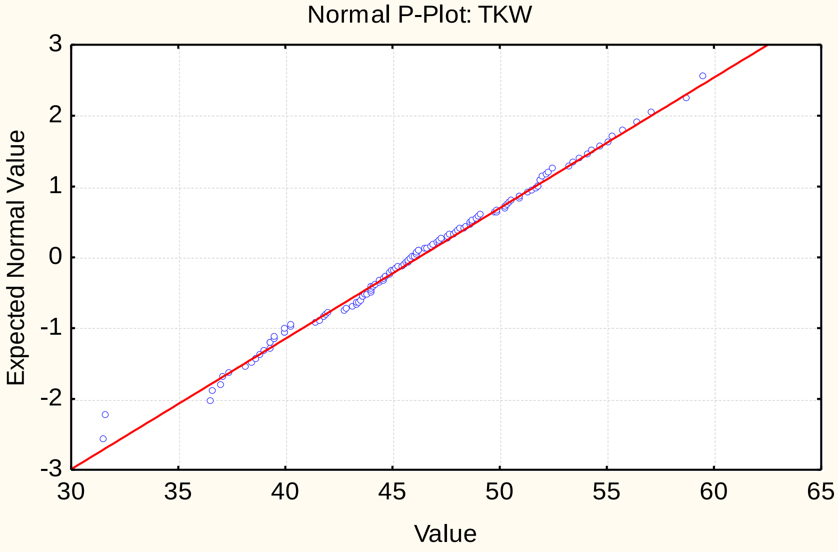

Summary Statistics:TKW
Valid N=128
Mean= 46,234180
Minimum= 31,500000
Maximum= 59,550000
Std.Dev.= 5,347910
Skewness= -0,066840
Kurtosis= 0,032543

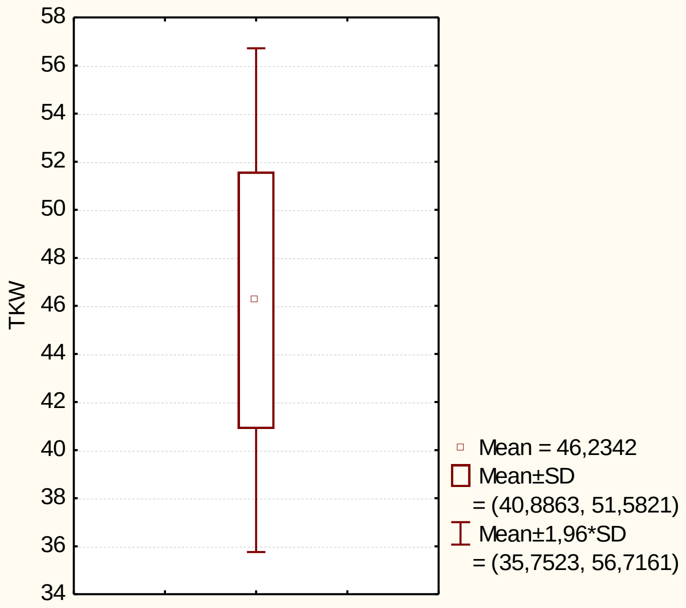

Supplement: Figure S3 — Phenotypic distribution for plant height (PH), heading date (HD), protein content (PC), and thousand kernel weight (TKW) in the durum sub-sample. (PDF) [file pone.0095211.s003.pdf]
