# Supplementary material for: Linkage Disequilibrium and Genome-Wide Association Mapping in Tetraploid Wheat (Triticum turgidum L.)
Source: PLoS One. 2014 Apr 23;9(4):e95211. doi: 10.1371/journal.pone.0095211 (PMC3997356; doi:10.1371/journal.pone.0095211)
Supplement: Table S1 — Overview of the LD in the intrachromosomal pairs for the whole collection, the durum sub-sample, and the two groups (Q1 and Q2). (DOCX) [file pone.0095211.s004.docx]

| **Dataset** | **Class** | **Total pairs** | **Mean *r^2^* of all pairs** | **N° significant pairs** | **Significant pairs (%)** | **N° physically linked pairs** | **Physically linked pairs (%)** | **Mean r^2^ for physically linked pairs** | **N° pairs in complete LD** |
| --- | --- | --- | --- | --- | --- | --- | --- | --- | --- |
| Whole | 1 | 2,717 | 0.26 | 2,111 | 77.7 | 1,649 | 60.7 | 0.41 | 190 |
| collection | 2 | 1,474 | 0.07 | 779 | 52.8 | 432 | 29.2 | 0.17 | 2 |
|  | 3 | 2,652 | 0.03 | 770 | 29.0 | 213 | 8.0 | 0.14 | 0 |
|  | 4 | 5,458 | 0.02 | 1,406 | 25.8 | 315 | 5.8 | 0.13 | 0 |
|  | **Total** | 12,301 | 0.08 | 5,066 | 41.2 | 2,609 | 21.2 | 0.31 | 192 |
| *Durum* | 1 | 2,717 | 0.37 | 2,149 | 79.1 | 2,004 | 73.7 | 0.49 | 277 |
| sub-sample | 2 | 1,474 | 0.13 | 809 | 54.9 | 683 | 46.3 | 0.25 | 2 |
|  | 3 | 2,652 | 0.05 | 758 | 28.6 | 485 | 18.3 | 0.17 | 0 |
|  | 4 | 5,458 | 0.03 | 854 | 16.6 | 471 | 8.6 | 0.13 | 0 |
|  | **Total** | 12,301 | 0.12 | 4,570 | 37.2 | 3,643 | 29.6 | 0.36 | 279 |
| Q1 | 1 | 2,635 | 0.38 | 2,146 | 81.4 | 1,814 | 68.8 | 0.53 | 278 |
|  | 2 | 1,410 | 0.14 | 806 | 57.2 | 594 | 42.1 | 0.28 | 2 |
|  | 3 | 2,553 | 0.06 | 778 | 30.5 | 361 | 14.1 | 0.20 | 0 |
|  | 4 | 5,123 | 0.03 | 888 | 17.3 | 282 | 5.5 | 0.17 | 0 |
|  | **Total** | 11,721 | 0.13 | 4,618 | 39.4 | 3,051 | 26.0 | 0.42 | 280 |
| Q2 | 1 | 2,476 | 0.20 | 937 | 37.8 | 743 | 30.0 | 0.58 | 199 |
|  | 2 | 1,374 | 0.05 | 228 | 16.6 | 132 | 9.6 | 0.25 | 2 |
|  | 3 | 2,442 | 0.05 | 404 | 16.5 | 242 | 9.9 | 0.26 | 0 |
|  | 4 | 4,745 | 0.04 | 677 | 14.3 | 256 | 5.4 | 0.19 | 0 |
|  | **Total** | 11,037 | 0.08 | 2,246 | 20.3 | 1,373 | 12.4 | 0.42 | 201 |

Genetic distances: class 1, <10 cM; class 2, 10-20 cM; class 3, 20-50 cM; class 4, >50 cM.

Mean allele frequency correlations (*r^2^*) for all pairs, number (N°) of pairs and percentage (%) significant in LD (*P*<0.01; *P*<0.001),

N° and % of physically linked pairs (*r^2^* > critical *r^2^*, *P*<0.01 and *P*<0.001), N° of pairs in complete LD (*r^2^* = 1).
